# Bacteria‐type‐specific biparental immune priming in the pipefish *Syngnathus typhle*

**DOI:** 10.1002/ece3.2391

**Published:** 2016-08-31

**Authors:** Anne Beemelmanns, Olivia Roth

**Affiliations:** ^1^ Helmholtz‐Centre for Ocean Research Kiel (GEOMAR)Evolutionary Ecology of Marine FishesDüsternbrooker Weg 2024105KielGermany

**Keywords:** Bacteria specificity, epigenetic inheritance, host–parasite interactions, nongenetic inheritance, transgenerational immune priming

## Abstract

The transfer of acquired and specific immunity against previously encountered bacteria from mothers to offspring boosts the immune response of the next generation and supports the development of a successful pathogen defense. While most studies claim that the transfer of immunity is a maternal trait, in the sex‐role‐reversed pipefish *Syngnathus typhle,* fathers nurse the embryos over a placenta‐like structure, which opens the door for additional paternal immune priming. We examined the potential and persistence of bacteria‐type‐specific parental immune priming in the pipefish *S. typhle* over maturation time using a fully reciprocal design with two different bacteria species (*Vibrio* spp. and *Tenacibaculum maritimum*). Our results suggest that *S. typhle* is able to specifically prime the next generation against prevalent local bacteria and to a limited extent even also against newly introduced bacteria species. Long‐term protection was thereby maintained only against prevailing *Vibrio* bacteria. Maternal and paternal transgenerational immune priming can complement each other, as they affect different pathways of the offspring immune system and come with distinct degree of specificity. The differential regulation of DNA‐methylation genes upon parental bacteria exposure in premature pipefish offspring indicates that epigenetic regulation processes are involved in transferring immune‐related information across generations. The identified trade‐offs between immune priming and reproduction determine TGIP as a costly trait, which might constrain the evolution of long‐lasting TGIP, if parental and offspring generations do not share the same parasite assembly.

## Introduction

On the strong selection imposed by parasites (Hamilton et al. 2008), hosts reacted with the evolution of highly specific immune systems (Schmid‐Hempel and Ebert 2003; Boots and Bowers 2004) that have the ability to differentiate among distinct parasite epitopes (Frank 2002; Kurtz 2005). Successful parasite clearance is the result of an interplay between genetic specificity and the phenotypic plastic immunological specificity. The latter (in vertebrates also called immune memory) permits a faster and more powerful immune response against previously encountered parasites (Kurtz 2005). To boost the immune system of the descendants, mothers can transfer this individual experience into the next generation (transgenerational immune priming [TGIP]) (Grindstaff et al. 2003; Little et al. 2003; Sadd et al. 2005; Grindstaff et al. 2006; Swain et al. 2006; Hasselquist and Nilsson 2009; Roth et al. 2009; Jiménez de Oya et al. 2011; Roth et al. 2012b; Ramos et al. 2014; Salmela et al. 2015). In vertebrates, TGIP is of particular importance for early life stages, as it bridges the maturation of the adaptive immune system that only starts after birth (Swain et al. 2002; Grindstaff et al. 2006; Swain et al. 2006; Zapata et al. 2006; Boulinier and Staszewski 2008; Hasselquist and Nilsson 2009; Zhang et al. 2013). With offspring development, TGIP declines (Lindholm et al. 2006), but its consequences can remain over several generations (Beemelmanns and Roth 2016 in review; Ismail et al. 2015; Norouzitallab et al. 2015).

While in most species (invertebrates and vertebrates) mothers deposit immunological substances directly into the eggs, species with some particular form of parental investment can additionally transfer their immunological experience during pregnancy and via breastfeeding or crop feeding (Patterson et al. 1962; Brambell 1970; Vandeputte‐Poma 1980; Reuman et al. 1983; Jacquin et al. 2012). As sperm were considered to be too small to deposit more than just the DNA (Wassarman et al. 2001) and fathers mostly lack a close physical connection to their offspring, TGIP was traditionally assumed to be limited to mothers. The recent discovery of paternal immune priming, both in invertebrates (Roth et al. 2010; Zanchi et al. 2011; Eggert et al. 2014) and in the vertebrate *Syngnathus typhle* (Roth et al. 2012b), emphasizes the importance of paternal effects (Crean and Bonduriansky 2014; Kaufmann et al. 2014). The sex‐role‐reversed pipefish *S. typhle* might be a unique case as males have an extraordinary close connection to their offspring during pregnancy and nurse their embryos over a placenta‐like structure (Roth et al. 2012b). This gives them the mechanistic opportunity to transfer immunological substances to their descendants. However, independent of a close physical connection, epigenetic marks can be passed on to the next generation (DNA‐methylation patterns and histone modifications) (Berger et al. 2009; Kappeler and Meaney 2010; Jablonka and Lamb 2015; Szyf 2015; Gapp and Miska 2016). Over their potential to modify offspring gene expression, these epigenetic marks may directly change the activity and specificity of offspring immune defense (Mukherjee et al. 2015) and facilitate the transfer of specific immune memory across generations (Youngblood et al. 2010; Gómez‐Díaz et al. 2012).

Just like a secondary encounter of a pathogen within lifetime induces immunological specificity, the transferred immunological information is also supposed to be specific to the parentally experienced pathogen genotypes (Little et al. 2003; Roth et al. 2009). Selection for pathogen‐specific TGIP is expected to be strongest when parents and offspring share the same environment and have overlapping generation times (Garnier et al. 2012). Being born in the parental environment thus enhances the probability to encounter the same pathogen epitopes repeatedly across generations due to the spatial heterogeneous distribution of pathogens (Dybdahl and Lively 1998; Lively and Dybdahl 2000). In migratory species without natal homing, the likelihood of repeated pathogen encounters across generations is lower, which should decrease selection for pathogen‐specific TGIP.

As induced immunity is costly due to a resource allocation trade‐off between immune response and other life‐history traits (development, maturation, reproduction, growth) (Lochmiller and Deerenberg 2000), the number of pathogens an individual can transfer specific immunity against is limited (Lochmiller and Deerenberg 2000; Schmid‐Hempel 2005; Ardia et al. 2011, 2012; Contreras‐Garduño et al. 2014). The probability of encountering the same pathogen both in the parental and in the offspring generation is thus supposed to influence the specificity, the intensity, and the length of a transgenerational immunization (Tidbury et al. 2011; Garnier et al. 2012).

The immune system of bony fishes (teleosts) characterizes a transition point between species relying exclusively on the phylogenetically conserved innate immune defense and species using a combination of innate and adaptive immunity (Flajnik and Kasahara 2010; Workenhe et al. 2010; Foey and Picchietti 2014). Due to their limited repertoire of antibodies and slow maturation of their lymphocytes, teleosts primarily rely on their innate immune defense (Uribe et al. 2011; Foey and Picchietti 2014). The survival of freshly hatched free‐living juveniles is enhanced by maternally derived immune components supplied during oogenesis such as antimicrobial peptides, lysosomes, complement components, lectins but also maternal antibodies (Bly et al. 1986; Sin et al. 1994; Hanif et al. 2004; Swain et al. 2006; Swain and Nayak 2009; Zhang et al. 2013). *Syngnathidae* (seahorses and pipefish) neither possess a spleen nor a gut‐associated lymphatic tissue, in which cells of the adaptive immune system assemble and proliferate (Matsunaga and Rahman 1998). The recent discovery of an absence of the MHC class II pathway represents a potential secondary reduction of the adaptive immune system (Haase et al. 2013). Due to this loss of a fundamental adaptive immune pathway, we aimed to investigate to what extent this fish species is able to transfer bacteria‐type‐specific immunity (specificity) from parents to offspring.

We assessed transgenerational bacteria‐type‐specific immune priming and maternal versus paternal specificity in offspring immune resistance using *S. typhle*. The parental generation was exposed to two different allopatric and heat‐killed bacteria epitopes (*Vibrio* spp. and *Tenacibaculum maritimum*) in a fully reciprocal mating design. We determined expression of 29 immune genes as well as immune cell activity of F1‐offspring (one‐week and four‐month‐old juveniles), exposed to the same (homologous) or the other bacteria isolate (heterologous) as their parents. This approach facilitated (i) the disentangling of the degree of parental bacteria‐type‐specific immune priming (specificity) over juvenile development and (ii) the extent of parental sex‐specific influences on different offspring immune pathways (innate and adaptive immune pathway, complement component system). To address the role of epigenetics in TGIP, we evaluated (iii) expression of genes associated with epigenetic regulation processes (DNA‐methylation and histone modifications). Finally, we investigated (iv) whether the channeling of energy resources to parental immune priming bears costs in terms of disadvantages in other life‐history traits.

## Material and Methods

### Parental generation (F0‐treatment)

**Figure 1:**
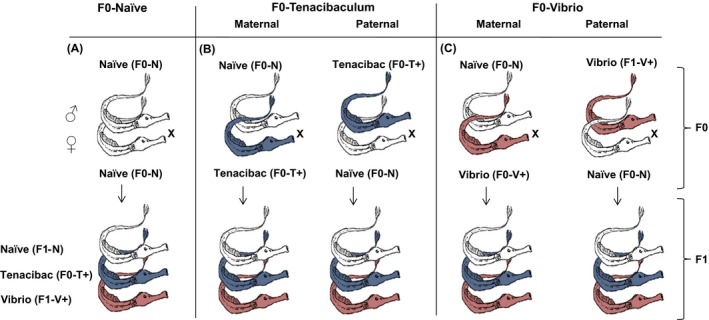
Experimental design to explore bacteria‐type‐specific immune priming in the pipefish *Syngnathus typhle* over one generation. In total, we analyzed 300 one‐week‐old and 90 4‐month‐old F1‐offspring of parental breeding pairs that received according to F0‐sex different F0‐bacteria treatments: (A) F0‐Naïve: no immune challenge for both parents (Naïve‐“F0‐N”); (B) F0‐Tenabibaculum: maternal immune challenge with *Tenacibaculum* (“Mat F0‐T+”); paternal: paternal immune challenge with *Tenacibaculum* (“Pat F0‐T+”); (C) F0‐Vibrio: challenge with *Vibrio* (“Mat F0‐V+”); paternal: paternal challenge with *Vibrio* (“Pat F0‐V+”). Each of the five parental treatment combinations was replicated four times resulting in 20 families per F0‐parental treatment group. F1‐offspring were exposed to the same heat‐killed *Vibrio* (“F1‐V+”) and *Tenacibaculum* (“F1‐T+”) bacteria species used for the parental generation or stayed without any treatment as control (“F1‐N”).

The parental pipefish generation was sampled, maintained, and treated as described in Beemelmanns and Roth (2016). Adult individuals received an injection with 50 *μ*L of 10^8^ cells/ml heat‐killed bacteria as immune challenge (Beemelmanns and Roth 2016). In our experimental design, always one sex of a mating pair was vaccinated with either *Vibrio* spp. (Italy species, I2K3) (Roth et al. 2012a) or *Tenacibaculum maritimum* (Suzuki et al. 2001). We applied immunologically novel (allopatric) bacteria strains to exclude any pre‐adaptation due to previous pathogen encounters in the wild. Upon immune challenge, the parental generation was kept in the following five final mating combinations (Fig. 1): (i) ♀Naïve × ♂Tenacibaculum, (ii) ♀Naïve × ♂Vibrio, (iii) ♀Tenacibaculum × ♂Naïve, (iv) ♀Vibrio × ♂Naïve, and (iv) ♀Naïve × ♂Naïve. The five parental treatment groups (F0‐bacteria) were replicated eight times, resulting in 40 breeding pairs (families). All couples mated successfully within one‐three days after the immune challenge and juveniles hatched after four weeks of male pregnancy. For further experimental work, we only included families with a minimum clutch size of 15 F1‐juveniles; we thus continued the experiment with F1‐individuals of 20 families.

### Filial generation 1 (F1‐treatment)

1‐week‐old offspring (8 days post birth) were exposed to the same heat‐killed *Vibrio* (V+) and *Tenacibaculum* (T+) bacteria species used for the parental generation or stayed without any treatment as control (*N*) (detailed description in Beemelmanns and Roth (2016)). For the F1‐bacteria treatment, we used 20 families with an equal distribution of four families per five F0‐bacteria treatments (Fig. 1). From each family, 15 offspring were randomly applied to the three F1‐bacteria treatments (five biological replicates per F1‐bacteria treatment) resulting in a total number of 300 juveniles. 1‐week‐old juveniles were pricked with a needle dipped in a solution containing 10^9^ cells/mL heat‐killed bacteria into the upper surface of the skin. After 20 h of incubation, their standard length was measured and whole‐body samples were used for RNA extraction (detailed description in Beemelmanns and Roth (2016)).

Remaining F1‐offspring were pooled within their parental treatment groups and transferred into 36 cm × 80 cm aquaria connected to a semi‐flow‐through circulation system using three tank replicates (density of 20 pipefish) per parental treatment for further rearing. For comparing TGIP effects between different maturation stages, four‐month‐old juveniles were exposed to the same procedure as the one‐week‐old juveniles, but injected intraperitoneally with 20 *μ*L 10^8^ cells/mL heat‐killed bacteria solution (F1‐V+, F1‐T+) or stayed naïve (F1‐N) using three biological replicates per F1‐bacteria treatment. In sum, 18 individuals of the five parental treatment groups were randomly collected out of the three tanks, resulting in a total number of 90 sampled juveniles**.** After incubation (20 h), body standard length and body mass were measured before the fish were sacrificed (detailed description in Beemelmanns and Roth (2016). Life‐history parameters (body size, mass, and liver weight) were collected, and a hepatosomatic index (HSI) was calculated as defined in Beemelmanns and Roth (2016). For characterizing the humoral innate and adaptive immune response, we measured the absolute number of lymphocytes and monocytes in the blood and head kidney according to the protocol of Roth et al. (2011).

As one‐week‐old juveniles were too small to dissect specific immune organs whole‐body samples were used for gene expression analysis, while for four‐month‐old juveniles, immunological active gill tissue was sampled. Accordingly, the RNA was extracted of 300 whole‐body samples of early‐stage juvenile pipefish (one week post birth) and 90 gill tissue samples of late‐stage juvenile pipefish (four months post birth). In the further analysis, tissue‐specific gene expression effects were taken into consideration.

The expression of 44 target genes and four housekeeping genes was measured for all 390 samples using a Fluidigm BioMark^™^ based on 96.96 dynamic arrays according to Beemelmanns and Roth (2016). The housekeeping genes ubiquitin (Ubi) and ribosome protein (Ribop) revealed the highest stability (geNorm M > 0.85), and their geomean was used to quantify relative gene expression of each target gene by calculating −∆Ct values (Beemelmanns and Roth 2016). We assessed target genes of following functional categories: (i) innate immune system, (ii) adaptive immune system, (iii) innate and adaptive immune genes, (iv) complement system, and (v) epigenetic modulators (DNA methylation, histone de/methylation, histone de/acetylation) (Beemelmanns and Roth 2016).

Remaining F1‐offspring were raised until they reached sexual maturity (approximately six‐seven months post birth) while they stayed without any immune treatment and time point of first reproduction was assessed. When F1‐individuals were sexually mature, they were crossed within the F0‐treatment groups and their clutch size was recorded.

### Data analysis and statistics

We evaluated whether gene expression (immune genes and epigenetic regulation genes), immune cell counts, and life‐history traits of juvenile pipefish from two consecutive age classes (one‐week‐old and four‐month‐old) revealed bacteria‐type‐specific effects upon the acute offspring exposure (“F1‐bacteria”) and the parental challenge (“F0‐bacteria”). Secondly, we explored whether offspring that received the same bacterial isolate as the parents (homologous) showed an enhanced immune response (immunological specificity) compared to those that experienced different bacteria exposures (heterologous) as their parents. To do so, we examined statistically and graphically the “F0‐bacteria” × “F1‐bacteria” challenge interaction. Thirdly, we analyzed parental sex‐specific (“F0‐sex”) immune priming differences to investigate whether mothers and/or fathers equally provide protection against previously encountered bacteria. For the identification of maternal and/or paternal immune priming specificity, we explored statistically the “F0‐bacteria” × “F1‐bacteria” × “F0‐sex” interaction term; family or tank was included as random term.

The data analysis was performed in R v 3.2.2 (R Core Team 2015) and PRIMERv6 (Clarke and Gorley 2006) according to Beemelmanns and Roth (2016). A permutational multivariate analysis of variance (PERMANOVA) was applied for immune gene expression (29 target genes) as well as epigenetic regulation genes (15 target genes) of one‐week‐old juveniles (300 samples) and four‐month‐old F1‐juveniles (90 samples). For the latter, we further assessed life‐history parameters (body size, body mass, hepatosomatic index (HSI)), and immune cell count measurements (lymphocyte/monocyte counts of blood and head kidney).

The PERMANOVA model (“vegan” package – “adonis” function in R) for each category was based on a Bray–Curtis matrix of nontransformed values in which we tested for the effects of “F0‐bacteria,” “F0‐sex,” and “F1‐bacteria” treatments and their interactions. The PERMANOVA was conducted by permuting treatments 1000 times and stratifying permutations within each family or tank replicate. To correct for the possible dependence between response variable and body size of the F1‐juveniles, we included standard length as a covariate in the PERMANOVA model. The analysis of similarity (ANOSIM) was performed with the software PRIMERv6 (Clarke 1993; Clarke and Gorley 2006) based on a Bray–Curtis distance matrix and 4th‐root transformation to disentangle differences between parental and offspring treatment groups using a pairwise comparison (Brazma and Vilo 2000). Further, we applied a between‐class analysis (BCA), which is a particular case of a principal component analysis (“ade4” package – “bca” function in R) to investigate graphical clustering according to the respective treatment group of interest (Dolédec and Chessel 1987; Thioulouse et al. 1997; Chessel et al. 2004). We performed a BCA of the gene categories of interest (immune genes, epigenetic genes) and immune cell count measurements. In addition, we evaluated the percentage of variance retained by the first two principal components (PCs) and calculated the variance explained by each response variable (gene contribution % to the total inertia) on PC1 and PC2. Genes with a contribution of above 25% summed average contribution were considered as “important genes” which added the highest variance to the dimensional space (Kassambara 2016). Further, we applied statistical univariate approaches for life‐history parameters and immune cell count measurements and focused on bacteria species‐specific immune priming effects. Hereupon, a linear mixed‐effect model was fitted for each response variable using the fixed interaction term “F0‐bacteria” × “F1‐bacteria”, while including family or tank as random term and implementing “size of juveniles” as a covariate. In addition, body size of F1‐juveniles was assessed separately as a phenotypic trait using the same model without a covariate. The linear mixed‐effect model was performed with the “lmer” function implemented in the “lme4” package of R (Bates et al. 2014) using type III sum of squares and Satterthwaite approximation for the degrees of freedom. All significant LMERs were followed by post hoc t‐tests applying the “ghlt” function associated in the “multcomp” package of R (Hothorn et al. 2008) for multiple comparisons of “F0‐bacteria” × “F1‐bacteria” interaction terms.

To assess life‐history traits of 6‐month‐old F1‐offspring (time point of maturity and clutch size), a linear mixed‐effect model (“nmle” package – “lme” function in R) according to Bates et al. (2014) was applied including the fixed factor “F0‐bacteria” and the random‐term “tank” in the model. Finally, a correlation analysis was applied to connect the biological relevance of gene expression patterns and immune parameters (“PerformanceAnalytics” package in R). Using a Pearson correlation matrix, we correlated each single gene (−∆Ct values) with each immune cell measurement in order to determine whether or not particular immune genes can be used as indicators for direct immune performance (Birrer et al. 2012).

## Results

### Bacteria‐type‐specific immune priming effect (F0‐bacteria treatment effect)

#### One‐week‐old F1‐juveniles: gene expression

**Figure 2:**
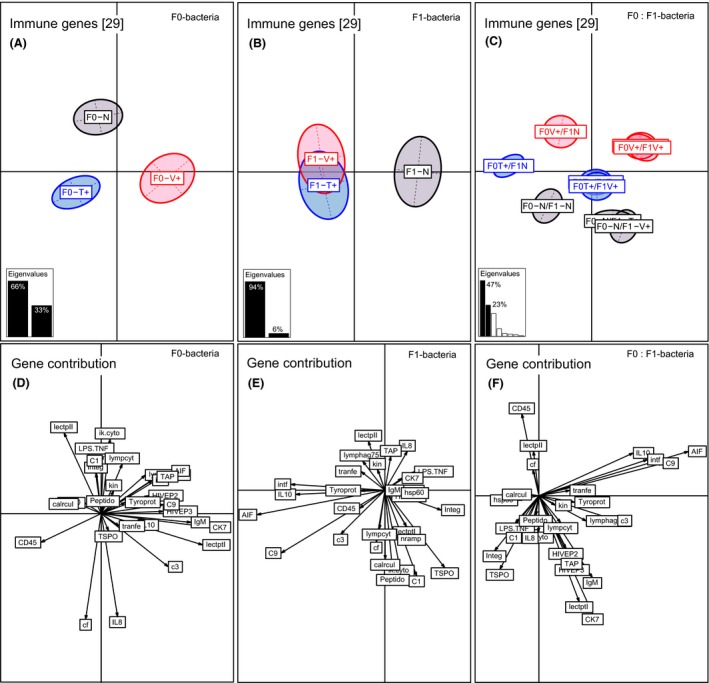
Between component analysis (BCA) based on 29 immune genes of one‐week‐old juveniles (*N* = 300). Different levels of factors were included in the between component analysis. (A) Factor F0‐bacteria treatment (F0‐Vibrio (F0‐V+) in red, F0‐Tenacibaculum (F0‐T+) in blue, F0‐Naïve (F0‐N) in black); (B) factor F1‐bacteria treatment (F1‐Vibrio (F1‐V+) in red, F1‐Tenacibaculum (F1‐T+) in blue, F1‐Naïve (F1‐N) in black); (C) factor F1:F0‐bacteria treatment interaction. In the underlying scatterplot (D‐F), the response variables (immune genes) are symbolized by arrows whereby the direction and the length of the arrows show the quality of the correlation between variables and principle components. The length of the arrow is directional proportional with the contribution of each variable (immune gene) to the total variability. The eigenvalues bar chart is drawn in the left corner, with the two black bars corresponding to the two axes used to draw the BCA plot.

Parental bacteria treatment (F0‐Vibrio or F0‐Tenacibaculum) changed the immune gene expression profiles in one‐week‐old F1‐offspring (PERMANOVA‐*immune F*_2,284_ = 10.21, *P* < 0.001; Table 1, Fig. 2A). On the BCA Axis 1 (66% variation), the two parental bacteria treatments clustered apart from the control on opposite sides, demonstrating a strong parental treatment effect (ANOSIM‐*immune* F0‐V+ vs. F0‐N *P* = 0.002; F0‐T+ vs. F0‐N *P* = 0.001; Table S1). Also, the two parental *Vibrio* and *Tenacibaculum* bacteria treatments significantly clustered opposed to each other (ANOSIM‐*immune* F0V+ vs. F0T+ *P* = 0.001; Table S1, Fig. 2A), leading to a triangle shape, representing a bacteria‐type‐specific immune priming effect. A similar pattern was identified for innate immune genes (PERMANOVA‐*innate F*_2,284_ = 11.88, *P* < 0.001; Table 1), innate and adaptive immune genes (PERMANOVA‐*innate & adaptive F*_2,284_=12.37, *P* < 0.001; Table 1), adaptive immune genes (PERMANOVA‐*adaptive F*_2,284_ = 7.42, *P* = 0.027; Table 1), and complement component genes (PERMANOVA‐*complement F*_2,284_ = 10.68, *P* < 0.001; Table 1). For the latter two gene categories, only the parental *Vibrio* treatment revealed a significant effect (ANOSIM‐*adaptive* F0‐V+ vs. F0‐N *P* = 0.003; ANOSIM‐*complement* F0‐V+ vs. F0‐N *P* = 0.006; Table S1). Immune genes explaining the *Vibrio*‐specific immune priming effect were chemokine 7 (17%), lectin protein I (15.5%), immunoglobulin light chain (12%), complement component 3 (6%), and HIVEP3 (6%) (Axis 1, 66%) (Table S3, Fig. 2D). In contrast, the following genes were driving the *Tenacibaculum*‐specific immune priming effect: CD45 (6%) (Axis 1, 66%) and coagulation factor II (19%), interleukin‐8 (18%), lectin II (11%) (Axis 2, 33%) (Table S3, Fig. 2D).

**Table 1:** Results from PERMANOVA of gene expression of one‐week‐old F1‐juveniles

One‐week‐old	Total	Residuals	F0‐bacteria				F1‐bacteria				F0‐sex				F0‐bacteria × F1‐bacteria			
	DF	Df	Df	F.Model	Pr(>*F*)	sig	Df	F.Model	Pr(>*F*)	sig	Df	F.Model	Pr(>*F*)	sig	Df	F.Model	Pr(>*F*)	sig
Immune genes [29]	299	284	2	10.21	**0.001**	***	2	6.63	**0.001**	***	1	5.76	**0.001**	***	4	0.82	ns	
Innate immune genes [13]	299	284	2	11.88	**0.001**	***	2	6.28	**0.001**	***	1	2.72	**0.001**	***	4	0.78	ns	
Innate & adaptive genes [5]	299	284	2	12.37	**0.001**	***	2	7.18	**0.001**	***	1	16.97	**0.001**	***	4	1.12	ns	
Adaptive immune genes [8]	299	284	2	7.42	**0.027**	*	2	1.16	0.059	•	1	3.54	**0.027**	*	4	0.65	ns	
Complement component genes [3]	299	284	2	10.68	**0.001**	***	2	19.40	**0.001**	***	1	2.56	**0.001**	***	4	1.28	ns	
Epigenetic genes [15]	299	284	2	1.77	**0.001**	***	2	2.04	**0.001**	**	1	5.68	**0.001**	***	4	0.89	ns	
DNA‐methylation genes [5]	299	284	2	1.50	**0.003**	**	2	1.28	**0.037**	*	1	11.03	**0.003**	**	4	0.67	ns	
Histone de/methylation genes [4]	299	284	2	1.34	**0.001**	***	2	2.67	**0.003**	**	1	2.63	**0.001**	***	4	0.79	ns	
Histone de/acetylation genes [5]	299	284	2	2.86	**0.001**	***	2	2.95	**0.001**	***	1	2.40	**0.001**	***	4	1.33	**0.006**	**

	Total	Residuals	F0‐bacteria × F0‐sex				F0‐sex × F1‐bacteria				F0‐bacteria × F1‐bacteria × F0‐sex				Size covariate			
	DF	df	Df	F.Model	Pr(>F)	sig	Df	F.Model	Pr(>F)	sig	Df	F.Model	Pr(>F)	sig	Df	F.Model	Pr(>F)	sig
Immune genes [29]	299	284	1	6.68	**0.001**	***	2	1.51	**0.002**	**	2	0.95	ns		1	4.27	ns	
Innate immune genes [13]	299	284	1	8.79	**0.001**	***	2	1.65	**0.003**	**	2	0.66	ns		1	5.99	ns	
Innate & adaptive genes [5]	299	284	1	7.16	**0.012**	*	2	1.15	ns		2	0.84	ns		1	3.35	**0.043**	*
Adaptive immune genes [8]	299	284	1	5.23	**0.005**	**	2	0.97	ns		2	1.17	**0.047**	*	1	1.31	ns	
Complement component genes [3]	299	284	1	0.94	**0.001**	***	2	2.05	**0.039**	*	2	1.00	ns		1	6.58	ns	
Epigenetic genes [15]	299	284	1	5.84	**0.001**	***	2	1.94	**0.003**	**	2	0.60	ns		1	6.06	ns	
DNA‐methylation genes [5]	299	284	1	1.56	**0.008**	**	2	2.20	**0.008**	**	2	0.68	ns		1	8.24	**0.035**	*
Histone de/methylation genes [4]	299	284	1	5.23	**0.005**	**	2	3.09	**0.005**	**	2	0.99	ns		1	7.93	**0.049**	*
Histone de/acetylation genes [5]	299	284	1	13.95	**0.003**	**	2	0.47	ns		2	0.34	ns		1	1.14	ns	

Multivariate PERMANOVA to assess the effect and interaction of the three fixed factors F0‐parents, F0‐sex, and F1‐offspring, size as covariate and family as strata term on relative gene expression values (−∆Ct values). Each analysis was based on a Bray–Curtis distance matrix with *P*‐values obtained by 999 permutations. Significant *P*‐values are marked in bold letters (significance code: <0.001***, 0.001**, 0.01*, 0.1> *P*‐value ≥0.05 trend •), whereas “ns” indicates no statistical difference.

**Figure 3:**
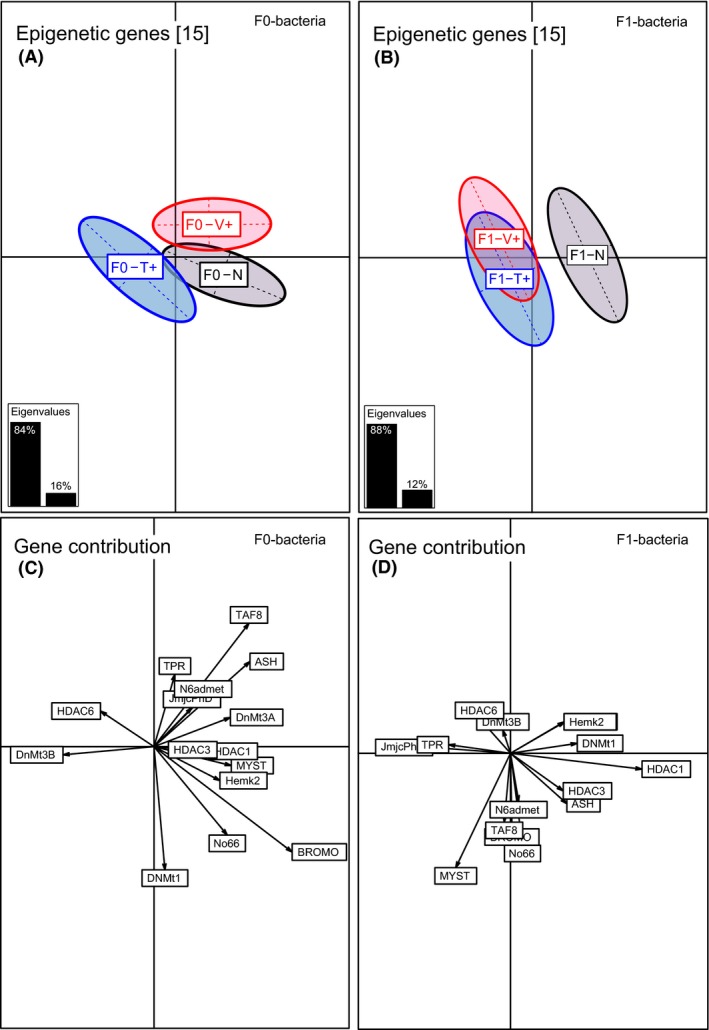
Between component analysis (BCA) based on epigenetic regultation genes of one‐week‐old juveniles (*N* = 300). Different levels of factors were included in the between component analysis. (A) Factor F0‐bacteria treatment (F0‐Vibrio (F0‐V+) in red, F0‐Tenacibaculum (F0‐T+) in blue, F0‐Naïve (F0‐N) in black); (B) factor F1‐offspring treatment (F1‐Vibrio (F1‐V+) in red, F1‐Tenacibaculum (F1‐T+) in blue, F1‐Naïve (F1‐N) in black). In the underlying scatterplots (C, D), the response variables (epigenetic regultation genes) are symbolized by arrows whereby the direction and the length of the arrows show the quality of the correlation between variables and principle components. The length of the arrow is directional proportional with the contribution of each variable to the total variability. The eigenvalues bar chart is drawn in the left corner, with the two black bars corresponding to the two axes used to draw the BCA plot.

Although genes associated with epigenetic regulation mechanism were differentially regulated upon parental immune challenge (PERMANOVA‐*epigen F*_2,284_ = 1.77, *P* < 0.001; Table 1, Fig. 3A), pairwise comparison between parental treatments solely revealed a significant difference between F0‐Vibrio and F0‐Tenacibaculum treatment, but no differences between F0‐bacteria treatments and F0‐naïve group (ANOSIM‐*epigen* F0‐V+ vs. F0‐T+ *P* = 0.001; Table S1). Epigenetic regulation genes with a high average contribution were histone acetyltransferase KAT2B (BROMO) (25%), transcription factor 8 (11%), histone methyltransferase (ASH2) (12%), DNA‐methyltransferase 3b (10%), DNA‐methyltransferase 3a (8%), lysine‐specific demethylase (No66) (7%), and histone acetyltransferase (7%) (Axis 1, 87%) (Table S5, Fig. 3C).

#### Four‐month‐old F1‐juveniles: gene expression

**Figure 4:**
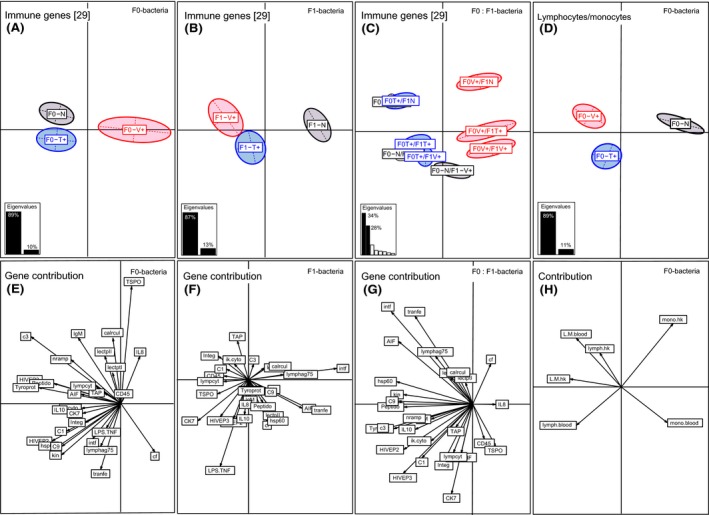
Between component analysis (BCA) based on 29 immune genes and on immune cell count measurements (lymphocyte and monocyte counts and ratio of head kidney and blood) in four‐month‐old juveniles (*N* = 90). Different levels of factors were included in the between component analysis. (A&D) Factor F0‐bacteria treatment (F0‐Vibrio (F0‐V+) in red, F0‐Tenacibaculum (F0‐T+) in blue, F0‐Naïve (F0‐N) in black); (B) factor F1‐bacteria treatment (F1‐Vibrio (F1‐V+) in red, F1‐Tenacibaculum (F1‐T+) in blue, F1‐Naïve (F1‐N) in black); (C) factor F1:F0‐bacteria treatment interaction. In the underlying scatterplots (E–H), the response variables (immune genes and immune cell measurements) are symbolized by arrows whereby the direction and the length of the arrows show the quality of the correlation between variables and principle components. The length of the arrow is directional proportional with the contribution of each variable to the total variability. The eigenvalues bar chart is drawn in the left corner, with the two black bars corresponding to the two axes used to draw the BCA plot.

In four‐month‐old juveniles, we found significantly altered expression profiles among the three parental bacteria treatment groups (PERMANOVA‐*immune F*_2,92_ = 4.90, *P* = 0.021; Table 2, Fig. 4A). In contrast to the results from one‐week‐old juveniles, the F0‐bacteria treatment effect is only preserved for the F0‐Vibrio challenge (ANOSIM‐*immune* F0‐V+ vs. F0‐N *P* < 0.001; Table S2). In the BCA, this is depicted by a sidewise‐shifted triangle shape, whereby only the parental F0‐Vibrio treatment group significantly clusters along the first axis (89% variation) opposed to the parental F0‐control group (Fig. 4A). In turn, the parental F0‐*Tenacibaculum* treatment did not influence the gene expression of four‐month‐old juveniles significantly (ANOSIM‐*immune* F0‐T+ vs. F0‐N *P* = 0.256; Table S2, Fig. 4A). This F0‐*Vibrio*‐specific parental immune priming effect was maintained by innate immune genes (PERMANOVA‐*innate F*_2,92_ = 3.14, *P* < 0.001, Table 2; ANOSIM‐*innate* F0‐V+ vs. F0‐N *P* = 0.005; Table S2). The following immune genes contributed to the *Vibrio*‐specific immune priming effect in four‐month‐old juveniles: complement component 3 (13%), tyroproteinkinase (11%), HIVEP3 (10%), HIVEP2 (8%), peptidoglycan recognition protein (7%), heat‐shock protein 60 (Hsp60) (6%), kinesin (6%), Nramp (4%), interleukin‐8 (5%) (Axis 1, 89%) as well as translocator protein (29%), transferrin (8%), calreticulin (8%), complement component 1 (6%), and immunoglobulin light chain (8%) (Axis 2, 10%) (Table S4, Fig. 4E).

**Table 2:** Results from PERMANOVA of gene expression of four‐month‐old F1‐juveniles

Four‐month‐old	Total	Residuals	F0‐bacteria				F1‐bacteria				F0‐sex				F0‐bacteria × F1‐bacteria			
	DF	Df	Df	F.Model	Pr(>F)	sig	Df	F.Model	Pr(>F)	sig	Df	F.Model	Pr(>F)	sig	Df	F.Model	Pr(>F)	sig
Immune genes [29]	107	92	2	4.90	**0.021**	*	2	3.65	**0.001**	***	1	1.63	**0.021**	*	4	0.82	ns	
Innate immune genes [13]	107	92	2	3.14	**0.001**	***	2	4.99	**0.001**	***	1	1.97	**0.001**	***	4	0.93	ns	
Innate & adaptive genes [5]	107	92	2	4.86	ns		2	5.02	**0.001**	***	1	5.91	ns		4	0.53	ns	
Adaptive immune genes [8]	107	92	2	3.74	ns		2	1.92	ns		1	0.16	ns		4	0.83	ns	
Complement component genes [3]	107	92	2	12.34	ns		2	0.26	ns		1	−0.02	ns		4	0.74	ns	
Epigenetic genes [15]	107	92	2	6.61	ns		2	1.21	ns		1	1.19	ns		4	0.80	ns	
DNA‐methylation genes [5]	107	92	2	4.30	**0.020**	*	2	1.27	ns		1	1.36	**0.020**	*	4	1.94	**0.012**	*
Histone de/methylation genes [4]	107	92	2	9.88	ns		2	1.70	0.085	●	1	0.97	ns		4	0.15	ns	
Histone de/acetylation genes [5]	107	92	2	6.00	ns		2	0.89	ns		1	1.63	ns		4	0.81	ns	
Immune cell counts [6]	107	72	2	12.38	**0.001**	***	2	3.82	**0.001**	***	1	1.33	**0.001**	***	4	0.43	ns	
Immune cell counts head kidney [3]	107	72	2	18.32	**0.001**	***	2	9.17	**0.001**	***	1	2.45	**0.004**	**	4	1.80	ns	
Immune cell counts blood [3]	107	72	2	9.83	**0.006**	**	2	3.22	**0.001**	***	1	1.62	**0.001**	***	4	0.23	ns	

	Total	Residuals	F0‐bacteria × F0‐sex				F0‐sex × F1‐bacteria				F0‐bacteria × F1‐bacteria × F0‐sex				Size covariate			
	DF	Df	Df	F.Model	Pr(>F)	sig	Df	F.Model	Pr(>F)	sig	Df	F.Model	Pr(>*F*)	sig	Df	F.Model	Pr(>*F*)	sig
Immune genes [29]	107	92	1	2.97	**0.022**	*	2	0.25	ns		2	1.23	ns		1	0.82	ns	
Innate immune genes [13]	107	92	1	1.08	**0.001**	***	2	0.30	ns		2	1.46	ns		1	0.52	ns	
Innate & adaptive genes [5]	107	92	1	4.40	ns		2	0.59	ns		2	0.98	ns		1	1.35	ns	
Adaptive immune genes [8]	107	92	1	8.01	ns		2	0.31	ns		2	2.12	**0.049**	*	1	0.61	ns	
Complement component genes [3]	107	92	1	0.32	ns		2	0.37	ns		2	0.66	ns		1	0.89	ns	
Epigenetic genes [15]	107	92	1	1.53	ns		2	0.48	ns		2	0.82	ns		1	0.69	ns	
DNA‐methylation genes [5]	107	92	1	1.56	**0.014**	*	2	0.94	ns		2	0.82	ns		1	0.34	ns	
Histone de/methylation genes [4]	107	92	1	1.27	ns		2	0.28	ns		2	1.51	ns		1	1.39	ns	
Histone de/acetylation genes [5]	107	92	1	1.85	ns		2	0.56	ns		2	0.68	ns		1	0.80	ns	
Immune cell counts [6]	107	72	1	1.29	ns		2	0.12	ns		2	1.19	**0.050**	*	1	1.23	ns	
Immune cell counts head kidney [3]	107	72	1	2.86	ns		2	2.20	ns		2	7.95	**0.009**	**	1	6.20	**0.003**	**
Immune cell counts blood [3]	107	72	1	0.69	ns		2	0.22	ns		2	0.21	ns		1	0.42	ns	

Multivariate PERMANOVA to assess the effect and interaction of the three fixed factors F0‐parents, F0‐sex and F1‐offspring, size as covariate and tank as strata term on relative gene expression values (−∆Ct values). Each analysis was based on a Bray–Curtis distance matrix with *P*‐values obtained by 999 permutations. Significant *P*‐values are marked in bold letters (significance code: <0.001***, 0.001**, 0.01*, 0.1> *P*‐value ≥0.05 trend ●), whereas “ns” indicates no statistical difference.

**Figure 5:**
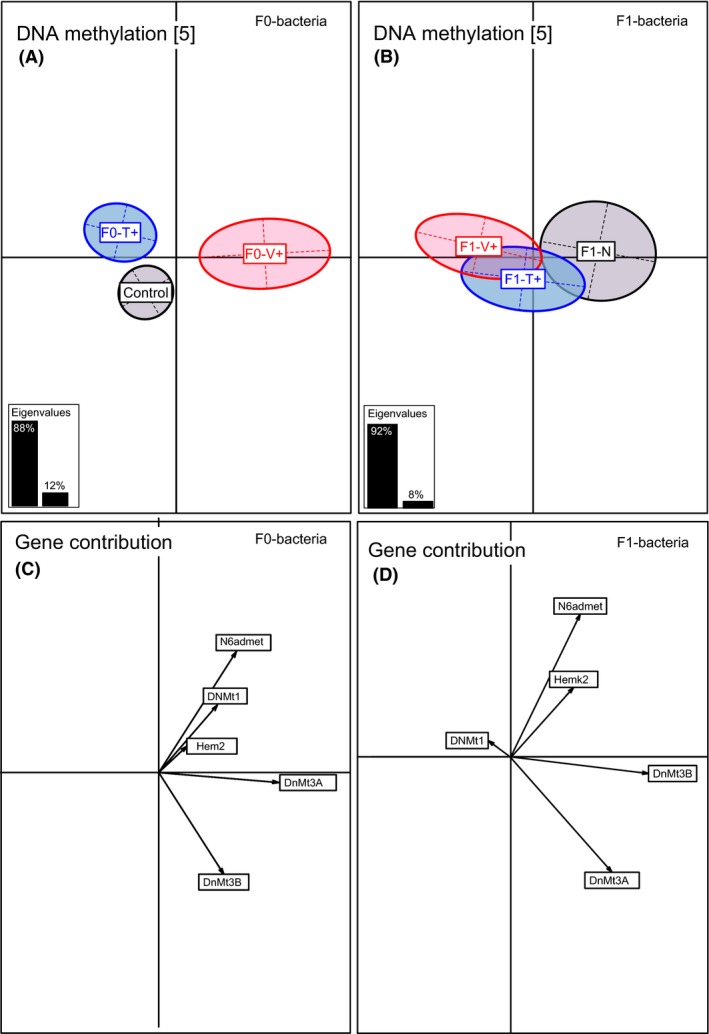
Between component analysis (BCA) based on 5 DNA‐methylation genes of four‐month‐old juveniles (*N* = 300). Different levels of factors were included in the between component analysis. (A) Factor F0‐bacteria treatment (F0‐Vibrio (F0‐V+) in red, F0‐Tenacibaculum (F0‐T+) in blue, F0‐Naïve (F0‐N) in black); (B) factor F1‐offspring treatment (F1‐Vibrio (F1‐V+) in red, F1‐Tenacibaculum (F1‐T+) in blue, F1‐Naïve (F1‐N) in black). In the underlying scatterplots (C, D), the response variables (DNA‐methylation genes) are symbolized by arrows whereby the direction and the length of the arrows show the quality of the correlation between variables and principle components. The length of the arrow is directional proportional with the contribution of each variable to the total variability. The eigenvalues bar chart is drawn in the left corner, with the two black bars corresponding to the two axes used to draw the BCA plot.

In four‐month‐old juveniles, solely DNA‐methylation genes were affected upon the F0‐bacteria treatment (PERMANOVA‐*DNA.methyl F*_2,92_ = 4.30, *P* = 0.020; Table 2) and displayed the same F0‐Vibrio‐specific pattern as previously described for innate immune genes (ANOSIM‐*DNA‐methyl* F0‐V+ vs. F0‐N *P* = 0.001; F0‐V+ vs. F0‐T+ *P* = 0.002; Table S2, Fig. 5A). *De novo* methyltransferases DNMT3a (61%) and DNMT3b (18%) (Axis 1, 86%) and DNMT1 (50%) and N6admet‐methylferase 36% (Axis 2, 13%) explained the highest variance (Table S6, Fig. 5C).

#### Four‐month‐old F1‐juveniles: immune cell counts

**Figure 6:**
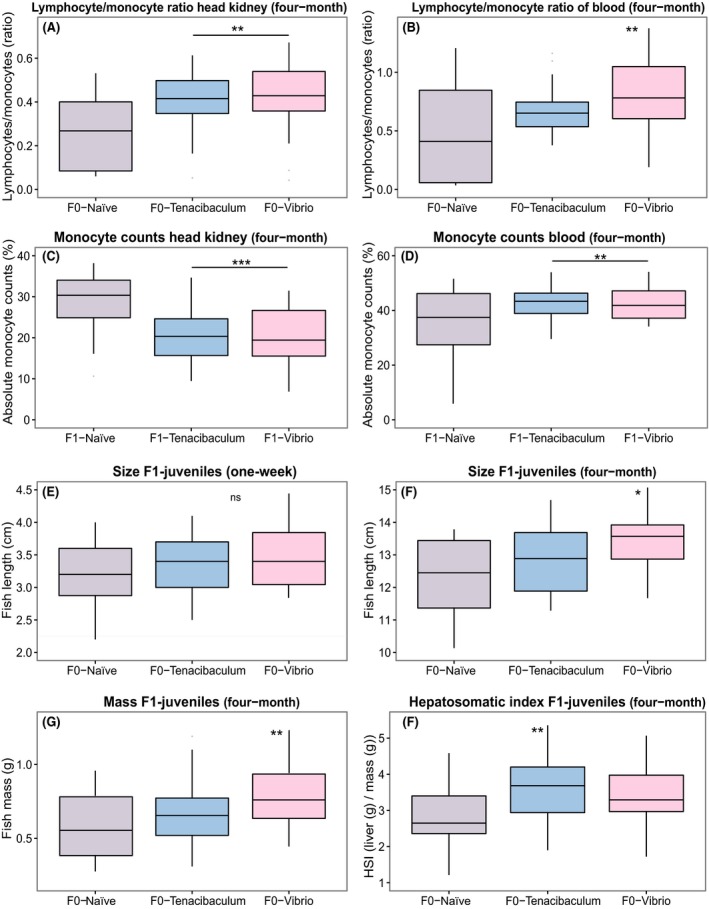
F0‐bacteria and F1‐bacteria treatment effects visualized by boxplots based on immune cell count measurements and life‐history parameter of four‐month‐old juveniles (*N* = 90) and size of one‐week‐old juveniles (*N* = 300). (A) F0‐bacteria treatment effects of lymphocyte/monocyte ratio of head kidney of four‐month‐old juveniles; (B) F0‐bacteria treatment effects of lymphocyte/monocyte ratio of blood of four‐month‐old juveniles; (C) F1‐bacteria treatment effects of monocyte counts of head kidney of 4‐month‐old juveniles; (D) F1‐bacteria treatment effects of monocyte counts of blood of four‐month‐old juveniles; (E) F0‐bacteria treatment effects of size of one‐week‐old juveniles, (F) F0‐bacteria treatment effects of size of four‐month‐old juveniles, (G) F0‐bacteria treatment effects of body mass (weight) of four‐month‐old juveniles, (H) F0‐bacteria treatment effects of hepatosomatic index (HSI) of four‐month‐old juveniles. Significance code: <0.001***, 0.001**, 0.01*. Abbreviation of F0 and F1‐bacteria treatments: Vibrio (F0‐V+) in red, F0‐Tenacibaculum (F0‐T+) in blue, F0‐Naïve (F0‐N) in grey. Depicted are the median, lower, and upper quartiles (box), and the minimum and maximum observed values (error bars).

The parental immune challenge of four‐month‐old F1‐offspring significantly affected the number of immune cells (lymphocytes and monocytes) in the head kidney and the blood (PERMANOVA‐immune‐cells F_2,72_ = 12.38, *P* < 0.001; Table 2, Fig. 4D, 4H). As demonstrated in the BCA, the two parental *Vibrio* and *Tenacibaculum* bacteria treatment groups were significantly clustering apart from the parental control group (ANOSIM‐immune‐cells F0‐T+ vs. F0‐N *P* = 0.001; F0‐V+ vs. F0‐N *P* = 0.001; Table S2, Fig. 4D) along the first axis (89% variation). The observed clustering pattern resembles a triangle shape, demonstrating a bacteria‐type‐specific immune priming effect based on immune cell production (ANOSIM *counts* F0T+ vs. F0V+ *P* = 0.001; Table S2, Fig. 4D). Using a statistical univariate approach, each cell count variable was analyzed separately in a linear mixed‐effect model (LMER) (Table 3). Particularly, we found an increased lymphocyte/monocyte ratio in the head kidney upon parental bacteria challenge (LMER‐*LM‐ratio.hk F*_2,34_ = 7.92, *P* = 0.001; *Tukey's HSD*: F0‐N < F0‐T+, F0‐N < F0‐V+; Table 3, Fig. 6A). The significantly higher proportion of lymphocytes in the blood of F1‐offspring with parental *Vibrio* challenge compared to the naïve control group (LMER‐*L/M‐ratio.blood F*_2,34_ = 5.40, *P* = 0.009; *Tukey's HSD*: F0‐N < F0‐V+; Table 3, Fig. 6B) indicates a higher humoral adaptive immune response specifically against parental *Vibrio* bacteria exposure.

**Table 3:** Results from univariate statistical analysis of life‐history parameters and immune cell count measurements in one‐week and four‐month‐old F1‐juveniles

	F0‐bacteria					F1‐bacteria				F0‐bacteria × F1‐bacteria			Size	
	NumDF = 2					NumDF = 2				NumDF = 4			NumDF = 4	
	F.value	Pr(>F)	Tukey's HSD			F.value	Pr(>F)	Tukey's HSD		F.value	Pr(>F)	Tukey's HSD	F.value	Pr(>*F*)
Size one‐week‐old	1.04	0.37	ns	ns	ns	2.30	0.10	ns	ns	1.96	ns			
Size four‐month‐old	4.41	**0.020**	F0N <F0V+	ns	ns	0.15	0.86	ns	ns	1.41	ns			
Mass four‐month‐old	6.02	**0.006**	F0N <F0V+	ns	F0T+<F0V+	1.22	0.30	ns	ns	1.90	ns		11.53	**0.001**
HSI four‐month‐old	7.82	**0.002**	(F0N <F0V+).	F0N <F0T+	ns	1.46	0.24	ns	ns	1.03	ns		11.22	**0.001**
Immune cell measurements four‐month‐old:														
Monocyte counts blood	5.39	**0.009**	F0N>F0V+	ns	ns	8.83	**<0.001**	F1N<F1V+	F1N<F1T+	6.54	**<0.001**	F0N/F1N versus F1V+ & F1T+	2.83	ns
Lymphocyte counts blood	3.51	**0.041**	F0N<F0V+	ns	ns	3.09	0.05	(F1N>F1V+)	(F1N> F1T+)	1.15	ns		0.52	ns
Lymphocyte/monocyte ratio blood	5.40	**0.009**	F0N<F0V+	ns	ns	12.91	**<0.001**	F1N>F1V+	F1N> F1T+	5.10	**0.001**	F0‐N/F1N versus F1V+ & F1T+	0.09	ns
Lymphocyte counts hk	0.52	ns	ns	ns	ns	6.62	**<0.001**	ns	F1N> F1T+	1.29	ns		10.83	**0.001**
Monocyte counts hk	25.24	**<0.001**	F0N>F0V+	F0N>F0T+	ns	19.00	**<0.001**	F1N>F1V+	F1N> F1T+	0.58	ns		3.97	**0.049**
Lymphocyte/monocyte ratio hk	7.92	**0.001**	F0N<F0V+	F0N<F0T+	ns	0.64	ns	ns	ns	3.67	**0.009**	F0V+/F1T+ versus F0V+/F1V+; F0T+/F1V+ versus F0V+/F1V+	1.27	ns

Linear mixed‐effect models (LMER) were applied to assess the effect and interaction of the two fixed factors F0‐bacteria and F1‐bacteria treatment, including size as covariate and family and/or tank as random term. LMER was performed with type III sum of squares and Satterthwaite approximation for degrees of freedom for each response variable. Tukey's HSD post hoc t‐test of the linear mixed‐effect model was performed with “lsmeans” to investigate pairwise comparison of corresponding levels of the fixed factors “F0‐bacteria” (parental control (F0N), parental *Vibrio* (F0V+) and *Tenacibaculum* (F0T+)) and “F1‐bacteria” (F1‐offspring naïve (F1N), F1‐offspring *Vibrio* (F1V+) and *Tenacibaculum* (F1T+)). Significant *P*‐values are marked in bold letters (significance code: <0.001***, 0.001**, 0.01*, 0.1> *P*‐value ≥0.05 trend ●). Response variables measured were body size of one‐week‐old and Four‐month‐old juveniles, for the latter also body mass, hepatosomatic index (HSI), lymphocyte and monocyte counts as well as their ratios of blood and head kidney (hk).

To connect the biological relevance of gene expression and cellular measurements, a correlation analysis was conducted for four‐month‐old F1‐individuals (−∆Ct values were correlated with cellular immune parameters). The following genes connected to pathways of the innate system positively correlate with the number of monocytes in the head kidney: *Lectin protein II* (*R* ^2^ = 0.26, *P* = 0.014), *interferon* (*R* ^2^ = 0.25, *P* = 0.019), *peptidoglycan* (*R* ^2^ = 0.30, *P* = 0.004), *tyroproteinkinase* (*R* ^2^ = 0.23, *P* = 0.032), *complement component 3* (*R* ^2^ = 0.35, *P* < 0.001) (Table S7). Additionally, the following immune genes displayed a positive correlation with the number of monocytes in the blood: *lectin protein I* (*R* ^2^ = 0.28, *P* = 0.038), *Ik‐cytokine* (*R* ^2^ = 0.23, *P* = 0.029), *complement component 3* (*R* ^2^ = 0.23, *P* = 0.01), *lymphocyte antigen 75* (*R* ^2^ = −0.22, *P* = 0.038), and *complement subcomponent 1q* (*R* ^2^ = 0.34, *P* = <0.001) (Table S7). Furthermore, there was a significant negative correlation between the number of lymphocytes and the expression of the adaptive immune genes *HIVEP3* (*R* ^2^ = −0.23, *P* = 0.031) and *complement subcomponent 1q* (*head kidney*:* R* ^2^ = −0.25, *P* = 0.016; *blood*:* R* ^2^ = −0.28, *P* = 0.007) (Table S7).

### Immune response against two different pathogens (F1‐bacteria effect)

#### One‐week‐old F1‐juveniles: gene expression

The acute immune challenge of one‐week‐old F1‐offspring (F1‐offspring treatment) significantly affected the expression of 29 immune genes (PERMANOVA‐*immune F*_2,284_ = 6.63, *P* < 0.001; Table 1, Fig. 2B). Here, the between‐class analysis (BCA) visualizes, that *Vibrio* (F1‐V+) and *Tenacibaculum* (F1‐T+) treatment groups cluster with overlapping centers of gravity opposed to the naïve (F1‐N) control group along the first axis, which explains 93% of the total variation (Fig. 2B). We did not find evidence for a bacteria‐type‐specific immune response as both treatment groups revealed an identical immune gene expression pattern (ANOSIM‐*immune* F1‐V+ vs. F1‐T+: *P* = 0.94; Table S1, Fig. 2B). Overall, innate immune genes (PERMANOVA‐*innate F*_2,284_ = 6.28, *P* < 0.001; Table 1), innate and adaptive immune genes (PERMANOVA‐*innate & adaptive F*_2,284_ = 7.18, *P* < 0.001; Table 1), and complement component genes (PERMANOVA‐*complement F*_2,284_ = 19.40, *P* < 0.001; Table 1) displayed a highly significant reaction as opposed to adaptive immune genes (PERMANOVA‐*adaptive F*_2,284_ = 1.16, *P* = 0.059; Table 1). The most important genes that were responsible for the F1‐bacteria treatment effect and can be considered as major drivers of immune response upon acute bacteria challenge are the following innate immune genes: Allograft inflammation factor (27%), complement component 3 (18%), interferon (15%), interleukin‐10 (13%), and translocator protein (6%) (Axis 1, 93%) (Table S3, Fig. 2E).

Besides, epigenetic genes revealed a treatment effect upon the acute immune treatment (PERMANOVA‐*epigen F*_2,284_ = 2.04, *P* = 0.001; Table 1, Fig. 3B). This was largely driven by histone de/acetylation genes (PERMANOVA‐de/*acetyl F*_2,284_ = 2.95, *P* < 0.001; Table 1), such as histone deacetylase 1 (HDAC1) (36%) (Axis 1, 83%) as well as histone acetyltransferase HAT1 (MYST) (31%), and histone acetyltransferase KAT2A (BROMO) (13%) (Axis 2, 16%) (Table S5, Fig. 3D).

#### Four‐month‐old F1‐juveniles: gene expression

The acute immune challenge of four‐month‐old F1‐offspring significantly affected the expression of 29 immune genes (PERMANOVA‐*immune F*_2,92_ = 3.65, *P* < 0.001; Table 2). In the corresponding between‐class analysis (BCA), *Vibrio* and *Tenacibaculum* treatment groups clustered without overlapping centers of gravities opposed to the naïve control group along the first axis, which explains 88% of total variation (Fig. 4B). As both F1‐treatment groups were statistically similar (ANOSIM‐*immune* F1‐V+ vs. F1‐T+ *P* = 0.24; Table S2, Fig. 4B), we could exclude a bacteria‐type‐specific immune response. The F1‐bacteria treatment response was predominantly driven by innate immune genes (PERMANOVA‐*innate F*_2,92_ = 4.99, *P* < 0.001; Table 2) and genes which are associated with innate and adaptive immune pathways (PERMANOVA‐*innate & adaptive F*_2,92_ = 5.02, *P* < 0.001; Table 2), whereas solely adaptive immune genes, complement component genes, and epigenetic genes were not affected (Table 2). Innate immune genes with a high contribution driving the immune response were interferon (27%), transferrin (16%), allograft inflammation factor (12%), and chemokine 7 (10%) (Axis 1, 87%) and lipopolysaccharide‐induced TNF‐*α* factor (27%) (Axis 2, 12%) (Table S4, Fig. 4F).

#### 4‐month‐old F1‐juveniles: immune cell counts

The humoral immune response measured through the absolute amount of immune cells in the head kidney (PERMANOVA‐*cells.hk F*_2,72_ = 9.17, *P* < 0.001, Table 2) and blood (PERMANOVA‐*cells.blood F*_2,72_ = 3.22, *P* < 0.001, Table 2) was activated upon the acute treatment in four‐month‐old F1‐offspring. More precisely, the amount of monocytes in the head kidney was significantly lower than in the naïve control group (LMER‐*mono.hk F*_2,66_ = 19.00, *P* < 0.001; *Tukey's HSD*: F1‐N > V+ and F1‐N > T+; Table 3, Fig. 6C) but in turn significantly higher in the blood (LMER‐*mono.blood F*_2,65_ = 8.83, *P* < 0.001; *Tukey's HSD*: F1‐N < F1‐V+ and F1‐N < F1‐T+, Table 3, Fig. 6D).

### Transgenerational bacteria specificity (F0‐bacteria** × **F1‐bacteria interaction)

#### One‐week and four‐month‐old F1‐juveniles: gene expression and immune cell counts

We examined statistically and graphically the F0‐bacteria × F1‐bacteria challenge interaction, whereby differences between homologous (parents and offspring received the same bacteria‐type) and heterologous (parents and offspring received different bacteria‐type) treatment combinations should indicate parental bacteria specificity effects across generations. However, based on all immune gene categories, the homologous (F0V+/F1V & F0T+/F1T+) and heterologous (F0V+/F1T+ & F0T+/F1V+) bacteria treatment combinations were not significantly different from each other and no significant interaction could be identified for both age categories (Figs. 2C and F, 4C and G, Table 1, 2). Univariate analysis of lymphocyte/monocyte ratio in the head kidney of four‐month‐old juveniles indicates a significant F0‐bacteria × F1‐bacteria interaction (LMER‐*L/M‐ratio.hk F*_4,66_ = 3.67, *P* = 0.009, Table 3) and displays a significant transgenerational *Vibrio* specificity effect (*Tukey's HSD*: F0‐V+/F1‐T+ vs. F0‐V+/F1‐V+, Table 3).

### Differences in maternal and/or paternal immune priming and maternal or paternal specificity effects

#### One‐week‐old F1‐juveniles: gene expression

A total of 29 immune genes of one‐week‐old F1 juveniles were strongly affected upon the F0‐paternal treatment than the F0‐maternal treatment (PERMANOVA‐*immune F*_1,284_ = 5.76, *P* < 0.001, Table 1; ANOSIM‐*immune* paternal vs. control *P* = 0.002; paternal vs. maternal *P* = 0.001, Table S1). Separated into functional immune gene categories, we found different intensities of maternal and paternal immune priming effects. Genes associated with the innate immune system were equally influenced by maternal and paternal bacteria treatment (PERMANOVA‐*innate F*_1,284_ = 2.72, *P* < 0.001, Table 1; ANOSIM‐*innate* paternal vs. control *P* = 0.001; maternal vs. control *P* = 0.001; paternal vs. maternal *P* = 0.003, Table S1). Genes of the adaptive immune system (PERMANOVA‐*adapt F*_1,284_ = 3.54, *P* = 0.027, Table 1; ANOSIM‐*adapt* paternal vs. control *P* = 0.046; paternal vs. maternal *P* = 0.003, Table S1) and complement component system (PERMANOVA‐*compl F*_1,284_ = 2.56, *P* = 0.001, Table 1; ANOSIM‐*adapt* paternal vs. control *P* = 0.041, Table S1) revealed solely F0‐paternal effects. Likewise, histone acetylation and deacetylation genes show significant F0‐paternal bacteria treatment influences (PERMANOVA‐*hist.de/acetyl F*_1,284_ = 2.40, *P* < 0.001, Table 1; ANOSIM‐*hist.de/acetyl* paternal vs. control *P* = 0.036; paternal vs. maternal *P* = 0.036, Table S1).

By analyzing the F0‐sex × F1‐bacteria as well as F0‐bacteria × F1‐bacteria × F0‐sex interaction terms, we were aiming to identify maternal and/or paternal bacteria specificity effects on F1‐offspring gene expression. Although innate immune genes (F0‐sex × F1‐bacteria, PERMANOVA‐*innate F*_1,284_ = 1.65, *P* = 0.003, Table 1), and adaptive immune genes (F0‐bacteria × F1‐bacteria × F0‐sex: PERMANOVA‐*adaptive F*_1,284_ = 1.75, *P* = 0.047, Table 1) display significant interaction terms, no traces for maternal nor paternal transfer of bacteria specificity could be identified (ANOSIM‐*innate* F0‐Mat/F1‐V+ vs. F0‐Mat/F1‐T+ *P* = 0.354; and F0‐Pat/F1‐V+, F0‐Pat/F1‐T+ *P* = 0.154; ANOSIM‐adaptive F0‐Mat/F0‐V+/F1‐V+ vs. F0‐Mat/F0‐V+/F1‐T+ *P* = 0.124 and F0‐Pat/F0‐V+/F1‐V+ vs. F0‐Pat/F0‐V+/F1‐T+ *P* = 0.154, Table S1).

#### Four‐month‐old F1‐juveniles: gene expression and Immune cell counts

In four‐month‐old juveniles, genes of the innate immune system were significantly influenced upon the F0‐paternal bacteria treatment (PERMANOVA‐*innate F*_1,92_ = 1.97, *P* < 0.001, Table 2; ANOSIM‐*innate* paternal vs. control *P* = 0.003; paternal vs. maternal *P* = 0.019, Table S2). In contrast, immune cell prevalence in the head kidney and blood was equally affected by both parents and no F0‐sex‐specific differences could be noticed (PERMANOVA‐*cell.counts F*_1,73_ = 1.33, *P* < 0.001 Table 2; ANOSIM‐*innate* paternal vs. control *P* = 0.001; maternal vs. control *P* = 0.001, Table S2). Similarly, DNA‐methylation genes were significantly influenced by both parents (PERMANOVA‐*DNA‐methyl F*_1,73_ = 1.36, *P* = 0.020, Table 2; ANOSIM‐*DNA‐methyl* paternal vs. control *P* = 0.003; paternal vs. maternal *P* = 0.003, Table S2). Adaptive immune genes showed a significant F0‐bacteria × F1‐bacteria × F0‐sex interaction (PERMANOVA‐*adaptive* F_1,74_ = 2.12, *P* = 0.049; Table 2), in accordance with immune cell measurements (PERMANOVA‐*immune.cells F*_1,74_ = 1.19, *P* = 0.042, Table 2). However, paternal bacteria specificity toward F0‐Vibrio bacteria was solely identified for immune cell count measurements in the head kidney (PERMANOVA‐*immune.cells.hk F*_1,74_ = 1.19, *P* = 0.042, Table 2; ANOSIM‐*immune.cells.hk*: F0‐Pat/F0‐V+/F1‐V+ vs. F0‐Pat/F0‐V+/F1‐T+ *P* = 0.035, Table S1).

### Costs of immune priming

#### One‐week‐old and four‐month‐old F1‐juveniles: Life history (size/weight/CF/HSI)

Whereas one‐week‐old F1‐offspring did not reveal a significant F0‐bacteria treatment effect on body size (LMER‐*size‐one‐week F*_2,17_ = 1.04, *P* = 0.365, Table 3, Fig. 6E), four‐month‐old F1‐offspring body length and mass was significantly influenced by the parental *Vibrio* immune challenge (LMER‐*size‐four‐month F*_2,33_ = 4.41, *P* = 0.020, Fig. 6F; LMER‐*mass‐four‐month* F_2,33_ = 6.02, *P* = 0.006, Fig. 6G, Table 3). four‐month‐old F1‐juveniles with parental *Vibrio* exposure were on average 1.03 (±0.3 s.e.) cm larger and 0.2 (±0.05 s.e.) g heavier compared to F1‐offspring of the F0‐control group (*Tukey's HSD‐size‐four‐month*: F0‐N < F0‐V+, Fig. 6G; *Tukey's HSD‐mass‐four‐month*: F0‐N < F0‐V+, F0‐T+ < F0‐V+, Fig. 6G, Table 3). Moreover, F0‐bacteria treatment of parents also affected the liver size of F1‐offspring (LMER‐*HSI‐four‐month F*_2,33_ = 7.82, *P* = 0.002, Fig. 6H, Table 3). Offspring with parental *Tenacibaculum* bacteria treatment had a significantly larger hepatosomatic index and offspring with parental *Vibrio* treatment a trend for a larger liver index in comparison with the control group (*Tukey's HSI‐four‐month*: F0‐N < F0‐T+, (F0‐N < F0‐V+ *P* = 0.05); Table 3, Fig. 6H).

#### Six‐month‐old F1‐juveniles: maturation

**Figure 7:**
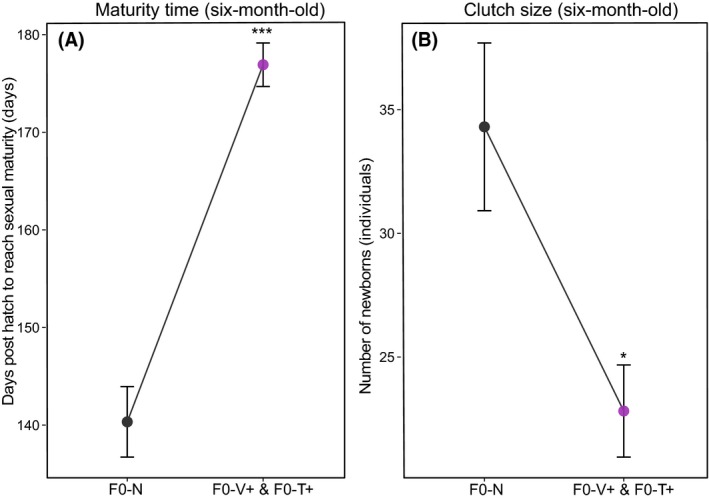
F0‐bacteria treatment on time for F1‐males to reach sexual maturity (A) and clutch size (B) of adult F1‐generation (six‐month‐old). Plots are depicted according to F0‐parental treatments (parental control (F0‐Naïve), and pooled parental bacteria challenged groups (F0‐Vibrio and F0‐Tenacibaculum). Respective error bars are representing standard error of the mean (±SEM).

Adult pipefish males (F1) of naïve parents (F0) developed about 36.5 (±1.5 s.e) days earlier in the season brood‐pouch tissue for sexual reproduction than offspring of parents with parental *F0‐Vibrio* and *F0‐Tenacibaculum* treatment (LME‐*maturity F*_1,126_ = 325, *P *< 0.001; Table S8, Fig. 7A). Adult offspring of the parental control group started to reproduce earlier and were having a significant higher number (11.5 ± 2.6 s.e individuals) of offspring per clutch (LME‐*clutch.size F*_1,15_ = 7.5, *P* = 0.025; Table S8, Fig. 7B).

## Discussion

### Bacteria‐type‐specific immune priming effects (Vibrio vs. Tenacibaculum)

Based on differential immune gene expression of 29 candidate genes and cellular immune response, our data indicates that the individual pathogen experience of pipefish parents influences the degree and strength of TGIP. Whereas both parental *Vibrio* and *Tenacibaculum* exposure induced the immune response of young juveniles, older juveniles (four‐month‐old) only displayed a parental immune priming effect against *Vibrio* bacteria. This indicates that the extent of parental bacteria‐type‐specific immune priming depends on the bacteria‐type applied and that its effect changes over the development of the descendants.

The genes contributing most to the variance of the transgenerational effect might be essential drivers of the bacteria‐type‐specific immune priming effect. In one‐week‐old juveniles, *Vibrio*‐specific immune priming was maintained by innate immune genes such as *lectin protein I*,* chemokine 7,* and *complement component 3*. All three genes code for innate immune proteins that act together for pathogen destruction over the complement system reacting via the lectin pathway and alternative pathway (Murphy 2011; Uribe et al. 2011). Here, the recognition and binding of bacteria cell‐wall‐associated carbohydrates over lectins or complement component 3 is followed by direct lysis over the membrane attack complex (MAC) but also a simultaneous secretion of signaling molecules (e.g., chemokine 7) which recruit and activate immune cells (e.g., macrophages) (Dodd and Drickamer 2001; Fujita 2002; Murphy 2011). Most likely the differential regulation of these genes implies an ongoing pathogen recognition followed by an immediate pro‐inflammatory response initiated 20 hours after the bacterial injection. Further, the expression of *HIVEP3* (V[D] J recombination of immunoglobulins and in MHC enhancer binding)*, immunoglobulin light chain* (recognition, opsonization and agglutination of pathogens) two genes associated with the antibody‐mediated adaptive immune pathway (Picchietti et al. 2006; Diepeveen et al. 2013), changed in case parents were exposed to a *Vibrio* challenge. This differential expression reflects the enhanced activation of adaptive immune components after parental *Vibrio* challenge and might reflect a potential transgenerational transfer of parental bacteria‐type‐specific immune memory. On the contrary, a central gene mediating the *Tenacibaculum*‐specific immune priming effect was pathogen recognition receptor *lectin type II*, which can function as an adhesion receptor but also as a phagocytic pathogen recognition receptor (Dodd and Drickamer 2001; Ewart et al. 2001; Fujita 2002). Similarly, the pro‐inflammatory signaling molecule *interleukin‐8* an important mediator for early attraction of neutrophil natural killer cells (phagocytosis, inflammatory activity), *coagulation factor II* responsible for a reduced flow draining to prevent distribution of pathogens, and *leukocyte common antigen CD45* regulating T‐cell and B‐cell antigen receptor signaling revealed a high importance. Likewise, as for the parental *Vibrio*‐specific immune priming effect, the *Tenacibaculum*‐specific response was influenced by genes essential for pathogen recognition and pro‐inflammatory response. However, *Vibrio*‐specific immune priming activated the complement component system and might explain the bacteria‐specific immune response due to the activation of different immune pathways in the one‐week‐old juveniles.

In four‐month‐old juveniles, a more diverse set of immune genes was differentially expressed upon parental *Vibrio* challenge and might be considered as essential players in *Vibrio*‐specific long‐term immune priming effect. Essential drivers were immune genes generating a pro‐inflammatory response such as *Peptidoglycan recognition proteins* (PGRPs) that recognize peptidoglycan on gram+ bacteria cell walls (such as *Vibrio* epitopes), revealing both peptidoglycan‐lytic amidase activity and broad‐spectrum bactericidal activity (Dziarski and Gupta 2006; Li et al. 2007); *translocator protein (TSPO)* crucial for immunomodulation like oxidative bursts by neutrophils and macrophages; *interleukin‐8 (IL‐8)* and *natural resistance‐associated macrophage protein (Nramp)* responsible for early attraction of neutrophil natural killer cells and activation of macrophages, but also *Tyroproteinkinase* critical in the cytokine receptor signaling pathways leading to T‐ and B‐cell activation (Murphy 2011; Uribe et al. 2011; Foey and Picchietti 2014). Moreover, *transferrin* is causing iron withholding a process preventing bacterial outgrowth (nutritional immunity), while *heat‐shock protein 60* chaperones assist in folding or unfolding of proteins and a central part of the general stress response (Murphy 2011; Uribe et al. 2011; Foey and Picchietti 2014). Similarly as for the *Vibrio*‐specific immune priming response in one‐week‐old juveniles, the complement component system was induced in four‐month‐old juveniles (*Complement components* 1 and 3) and identical genes of the adaptive immune pathway (HIVEP2 & HIVEP3 and immunoglobulin light chain) that were driving the *Vibrio*‐specific long‐term immune priming effect. On top of that the parental *Vibrio* challenge induced a significantly higher lymphocyte/monocyte ratio in the blood of four‐month‐old F1‐juveniles in comparison with the parental *Tenacibaculum* treatment which also revealed a certain degree of *Vibrio* specificity. This suggests that parents specifically transferred protective cues against *Vibrio* bacteria, leading to long‐term stimulation effects on offspring immunity, potentially enhancing the immune performance of their offspring.

*Vibrio* bacteria are the most abundant and diverse opportunistic pathogens in the marine realm (Frédérique Le Roux et al. 2015). They occur on a continuum from pathogenic over opportunistic to symbiotic or commensal and can be isolated from the organs of the broad‐nosed pipefish *S. typhle* (Roth et al. 2012a), but can also be found free‐living in the marine environment (Frédérique Le Roux et al. 2015). As such, the wild‐caught parental generation had already encountered a diversity of different *Vibrio* phylotypes in the field (Roth et al. 2012a). Also in this experiment, even though we filtered the water in the aquaria to prevent confounding effects with other bacterial infections, we could not exclude that the parental and the F1‐generation were in contact with Baltic *Vibrio* bacteria species throughout the experiment. To exclude a previous immunological encounter with the experimental *Vibrio* phylotype, we used an allopatric *Vibrio* isolate of an Italian pipefish (Italy‐strain I2K3) (Roth et al. 2012a). In a previous study, we could show that bacteria assemblies are distinct among pipefish populations and that the antimicrobial activity of Baltic pipefish is lower against allopatric *Vibrio* Italy strains in comparison with sympatric Baltic Vibrio strains (Roth et al. 2012a). Our current results may suggest a robust *Vibrio*‐specific immune priming effect, which implies that the parental generation created an immune memory against Italian *Vibrio* bacteria and transferred long‐lasting cues to the next generation. The flagellum of pathogenic *Vibrio alginolyticus* bacteria triggers a specific Toll‐like receptor (TLR5) that is followed by a signal cascade over Nk‐transcription factor and a pro‐inflammatory immune response (Wang et al. 2016). We may speculate that the flagellum structure of the applied *Vibrio* strain (I2K3) could have been similar to local *Vibrio* phylotypes of their natural habitat or prevalent *Vibrio* phylotypes during the experiment and therefore, was more familiar for the immune system of this pipefish population. Even if flagella structures of Italian and Baltic *Vibrio* were distinct and another mechanism may explain the observed pattern, we here identified that the offspring received non‐genetic information about European *Vibrio* bacteria from their parents. Within the four months of the experiment, the juvenile pipefish started to develop a specific immune response against *Vibrio* bacteria, which suggests bacteria‐type‐specific TGIP.

In contrast, the *Tenacibaculum maritinum* bacteria used in this experiment were isolated from a pacific seabream species (Suzuki et al. 2001). Although we cannot exclude the possibility that the wild parental pipefish population were in contact with *Tenacibaculum* bacteria in the Baltic Sea (Frette et al. 2004), we presumed that this bacterium isolate was immunologically novel for the Baltic pipefish. As the parental long‐term immune priming against *Tenacibaculum* bacteria was significantly reduced in four‐month‐old juveniles, it strongly indicates that immune priming against newly introduced and rare bacteria is decreasing faster during development (Lindholm et al. 2006; Wilson and Réale 2006). Hence, based on our results, it is tempting to speculate that immune priming against prevalent and more familiar bacteria, with which the parental population was repeatedly in contact before, is more pronounced because the likelihood of a secondary exposure is high. Consequently, the diversity and quantity of bacteria‐type‐specific immune transmission to offspring is reflecting the differences in pathogen environment experienced by their parents as it was shown for vertebrates of higher phylogenetic order, for example, specific antibody transmission in birds (Grindstaff et al. 2006). To finally assess this, pipefish of different populations that encountered a diverse set of bacteria during the last generations would need to be assessed in a similar experiment.

### Parental sex‐specific effect (maternal versus paternal effects)

As pipefish females invest into the eggs, and males potentially prime the immune system of their offspring via the placenta‐like structure during male pregnancy, shared tasks in immunological transfer between males and females may have evolved. Our results suggest that a dissimilar extent of maternal and paternal influences on different offspring immune pathways has evolved to reach an optimal immune protection. In general, expression of immune genes in one‐week‐old F1‐juveniles was predominantly influenced by the paternal bacteria treatment. Likewise, innate immune genes of four‐month‐old juveniles were only affected upon the paternal treatment, and F1‐offspring receiving a homologous *Vibrio* bacteria challenge as their fathers showed an induced immune cell activity in the head kidney, indicating the transfer of paternal *Vibrio* specificity (Beemelmanns and Roth 2016). Males may transfer information about immediate protection cues against prevalent pathogens in their environment through the placenta‐like structure during male pregnancy and/or through epigenetic marks. As offspring are born in their father's environment and most probably experience a similar pathogen assembly, selection could favor the paternal transfer about the local parasitic environment to provide a solid long‐term protection. In case these pathogens are encountered during the next generation, paternal TGIP is adaptive as it will increase the fitness of the fathers (Crean and Bonduriansky 2014).

Teleost females prime the immune system of their offspring by the deposition of immunoglobulins, complement components, antimicrobial peptides, lectins, and corresponding mRNA transcripts through the yolk into the eggs (Magnadottir et al. 2005; Picchietti et al. 2006; Swain et al. 2006; Swain and Nayak 2009; Zhang et al. 2013). Maternal immune priming differentially regulated only innate immune gene expression of one‐week‐old juveniles, and even this effect faded with offspring development. As the affected immunological pathways are parent‐specific, maternal and paternal immune priming can complement each other. This gives biparental TGIP a double benefit that could even be more than additive, as immunity is transgenerationally provided against specific local bacteria species that either mothers or fathers have previously encountered (Roth et al. 2012b). This could result in an enhanced phenotypic plastic immune response with the potential to induce a more specific and stronger reaction upon local and prevalent pathogens. Consequently, maternally and paternally inherited bacteria‐type‐specific immune priming is thus not only providing specific protection for the young progeny, but it also allows organisms to plastically adapt to the prevailing pathogen environment (Little et al. 2003; Moret 2006; Roth et al. 2012b).

### Transmission of parental bacterial specificity (F1‐treatment and interaction)

1‐week‐old and four‐month‐old juveniles upregulated the same set of immune genes, independent of which bacterium they were exposed to. In four‐month‐old juveniles, 20 h after the immune challenge monocytes already migrated from the head kidney through the bloodstream to elicit an inflammation response in peripheral organs (Janeway et al. 2008; Murphy 2011). Further, we found a positive correlation between innate immune genes (*lectin protein II, lectin protein I, complement component 1 and 3, interferon, peptidoglycan recognition protein, tyroproteinkinase, Ik‐cytokine*) and amount of monocytes. This verifies a direct connection between gene activity and innate immune performance in accordance with a previous study (Birrer et al. 2012). However, lymphocytes, cells of the adaptive immune system responsible for generating a highly specific antibody‐mediated response and the elimination of specific pathogens, were not significantly influenced upon the direct treatment. Yet, certain adaptive immune genes displayed a positive correlation (*HIVEP3* and *lymphocyte antigen 75)* with lymphocytes in the head kidney and blood. Likewise lymphocyte/monocyte ratio of the head kidney revealed paternal *Vibrio* specificity effects, indicating that the adaptive immune system started to be active. Hence, the incapacity to create immune specificity upon parental homologous bacteria exposure as verified in the immune gene expression level might be explained either by the nonfully activated adaptive immune system, or even could be ascribed to abnormalities of the pipefish adaptive immune system (Haase et al. 2013). *Syngnathus typhle* not only lacks a spleen in which antibody producing T‐cell and B‐cell assemble and proliferate but also the MHCII machinery and T‐cell‐related genes like CD8*β*/TCR*γ*, known to be key innovations of the adaptive immune system, were secondarily lost (Matsunaga and Rahman 1998; Haase et al. 2013).

### Mechanism of immune priming (epigenetic regulation genes)

To advance our aim to pinpoint the underlying mechanism of TGIP, we analyzed genes responsible for epigenetic regulation processes that can indirectly affect the transcriptional regulation of immune gene expression. In 1‐week‐old juveniles, the expression of histone acetylation and deacetylation genes was influenced by the bacteria exposure of the fathers. As histone modifications are important modulators of innate immune memory of macrophages (Netea et al. 2015, 2016) and heritable across generations (Campos et al., 2014; Gaydos et al., 2014; Jones, 2015), histones might also act as “carriers of epigenetic information” for pathogen experiences (Ragunathan et al., 2015) and are potentially involved in paternal transgenerational immune priming.

In four‐month‐old juveniles, genes responsible for DNA methylation such as *DNMT 3a* and *DNMT 3b* showed a strong impact upon the parental bacteria treatment. Whereas maintenance DNA‐methyltransferase *DNMT1* copies complementary marks of newly replicated DNA (Bestor, 2000), *DNMT 3a* and *DNMT 3b* conduct de novo new chemical modifications, which are essential for epigenetic changes based on environmental stress (Okano et al., 1999; Mitchell et al., 2014) and therefore might be important regulators. Equal maternal and paternal treatment effects on DNA‐methylation genes were found in four‐month‐old juveniles that can even persist to the second generation (Beemelmanns and Roth 2016 in review). As these crucial regulation genes of the transcriptional reprogramming were significantly affected by the parental *Vibrio* treatment, our results point to a potential connection of transgenerational immune priming to epigenetic inheritance. The altered expression of genes coding for key players in the epigenetic regulation machinery of immune gene expression supports our hypothesis that epigenetic processes are involved in bacteria‐type‐specific immune priming.

### Energetic costs of bacteria‐type‐specific immune priming

Parental *Vibrio* challenge not only induced offspring immune response but also accelerated their growth and weight increase, an effect that was identified one week after birth already, but consisted to four months post birth. While an efficient specific immune defense and a faster development can be advantageous, they are also costly in terms of energy resources, particularly if the parasitic environment is not met in the next generation. Most likely, the benefits are in such a scenario traded off against other fitness parameters (Lochmiller and Deerenberg 2000; Ardia et al. 2012). The liver, an important storage organ of energy reserves, served as estimate about the metabolism and energy status of the fish (Chellappa & Huntingford 1995). Both parental bacteria treatments positively affected the hepatosomatic index, suggesting that immune primed offspring revealed a better metabolic status. However, costs were found later during sexual maturation of the F1‐adults. Prolonged time of males to develop a brood‐pouch tissue and reach sexual maturation delayed reproduction period of about one month and an overall significant smaller clutch size compared to offspring without parental bacteria challenge. This confirms that immunological costs were compensated by reduced energy investment into reproduction. A significant shift of maturation time and reproduction would have essential ecological consequences for the pipefish. Every summer season (April/May), the pipefish population migrates to the seagrass meadow of the Baltic coastlines where males can reproduce up to four times per season with several females (Berglund et al. 1986, 1989; Berglund 1993). Therefore, it is advantageous to mate as early as possible in the season due to predator pressure of a new habitat and also due to the polyandrous mating behavior (Berglund 1993). Hence, channeling the resources toward more efficient immunity and balancing these benefits with reduced reproduction might be a costly strategy, which may shape the outcome of immune priming across generation (Contreras‐Garduño et al. 2014). While bacteria‐type‐specific biparental immune priming in the pipefish might be beneficial on the individual level, it could have severe ecological and evolutionary consequences on the population level and may alter the dynamics of host/pathogen interactions (Mostowy et al. 2012; Tate and Rudolf 2012). When it imposes costs in terms of reduced reproduction, it can increase parasite prevalence, might lead to a pronounced destabilization effects on host–parasite dynamics, and change the spread of epidemics in a population (Tidbury et al. 2011; Mostowy et al. 2012; Tate and Rudolf 2012; Tidbury et al. 2012).

Nevertheless, selection for immune priming indicates that there must be an adaptive net influence especially when there is a high probability of encountering the same pathogen both in the parental and the offspring generation and that total benefits will outweigh the associated costs (Schmid‐Hempel 2011; Kaufmann et al. 2014). Apart from higher immunity, also other benefits like a larger body size, increased weight and better metabolic condition were identified, which could at least partly compensate the costs of reduced reproduction. Thus, producing fewer offspring in a good shape might be a better strategy. The latter not only permits the parental transfer of specific protection to the offspring, but it also allows organisms to plastically adapt to the prevailing pathogen environment.

## Conflict of Interest

None declared.

## Supporting information


**Table S1** Results from PERMANOVA and ANOSIM analysis of one‐week‐old F1‐juveniles per functional gene categories.
**Table S2** Results from PERMANOVA and ANOSIM analysis of four‐month‐old per functional gene categories and immune cell measurements.
**Table S3** Immune gene contribution (29) of one‐week‐old juveniles based on the scores of two extracted principle coordinates
**Table S4** Immune gene contribution (29) of four‐month‐old juveniles based on the scores of two extracted principle coordinates.
**Table S5** Epigenetic gene contribution (15) of one‐week‐old juveniles based on the scores of two extracted principle coordinates.
**Table S6** DNA‐methylation gene contribution (5) of four‐month‐old juveniles based on the scores of two extracted principle coordinates.
**Table S7** Linear Mixed effect model to test for F0‐bacteria effects in time of maturation of adult pipefish males and clutch size of six‐month‐old F1‐offspring.
**Table S8** Correlation analysis between immune genes and monocyte and lymphocyte count measurements from four‐month‐old F1‐offspring.
