# Supplementary material for: Bacteria‐type‐specific biparental immune priming in the pipefish Syngnathus typhle
Source: Ecol Evol. 2016 Aug 31;6(18):6735–57. doi: 10.1002/ece3.2391 (PMC5058542; doi:10.1002/ece3.2391)
Supplement: Supplementary file 1 — Table S1 Results from PERMANOVA and ANOSIM analysis of one‐week‐old F1‐juveniles per functional gene categories. Table S2 Results from PERMANOVA and ANOSIM analysis of four‐month‐old per functional gene categories and immune cell measurements. Table S3 Immune gene contribution (29) of one‐week‐old juveniles based on the scores of two extracted principle coordinates Table S4 Immune gene contribution (29) of four‐month‐old juveniles based on the scores of two extracted principle coordinates. Table S5 Epigenetic gene contribution (15) of one‐week‐old juveniles based on the scores of two extracted principle coordinates. Table S6 DNA‐methylation gene contribution (5) of four‐month‐old juveniles based on the scores of two extracted principle coordinates. Table S7 Linear Mixed effect model to test for F0‐bacteria effects in time of maturation of adult pipefish males and clutch size of six‐month‐old F1‐offspring. Table S8 Correlation analysis between immune genes and monocyte and lymphocyte count measurements from four‐month‐old F1‐offspring. [file ECE3-6-6735-s001.docx]

# Appendix (Supplemental Material)

**Table S1: Results from PERMANOVA and ANOSIM analysis of one-week-old F1-juveniles per functional gene categories.** Multivariate ANOSIM was performed following significant PERMANOVA effect to assess differences in the gene expression profiles per treatment groups applying pairwise comparison on relative gene expression data (−∆Ct-values) based on a Bray-Curtis distance matrix and 999 permutations. Pairwise comparison was conducted for following fixed factors and their interaction: F0-bacteria treatment effect (parental control (F0N), parental *Vibrio* (F0V+) vs *Tenacibaculum* (F0T+)), F0-sex´ (maternal (F0Mat), paternal (F0Pat) vs control (F0N)) and `F1-bacteria´ (F1-offspring control (F1N), F1-offspring *Vibrio* (F1V+) and *Tenacibaculum* (F1T+)).

| **One week-old** | **Immune genes (29)** | **Innate genes (13)** | **Innate & Adaptive genes (5)** | **Adaptive genes (8)** | **Complement component genes (3)** | **Epigenetic genes (15)** | **DNA-methylation genes (5)** | **Histone de/methylation genes (4)** | **Histone de/acetylation**  **genes (5)** |
| --- | --- | --- | --- | --- | --- | --- | --- | --- | --- |
| **F0-bacteria** | **<0.001***** | **<0.001***** | **<0.001***** | **0.026*** | **<0.001***** | **<0.001***** | **0.002**** | **<0.001***** | **<0.001***** |
| **Global R** | **0.108** | **0.110** | **0.033** | **0.076** | **0.038** | **0.006** | **0.005** | **0.006** | **0.001** |
| **Significance level** | **0.001** | **0.001** | **0.001** | **0.001** | **0.001** | **0.308** | **0.328** | **0.304** | **0.133** |
| **Groups** |  |  |  |  |  |  |  |  |  |
| **F0V+, F0T+** | **0.001** | **0.001** | **0.001** | **0.001** | **0.001** | **0.01** | **0.002** | **0.04** | **0.05** |
| **F0V+, F0N** | **0.002** | **0.001** | ns | **0.003** | **0.006** | ns | ns | ns | ns |
| **F0T+, F0N** | **0.001** | **0.001** | ns | ns | Ns | ns | ns | ns | ns |
| **F0-sex** | **<0.001***** | **<0.001***** | **<0.001***** | **0.026*** | **<0.001***** | **<0.001***** | **0.002**** | **<0.001***** | **<0.001***** |
| **ANOSIM-Global R** | **0.052** | **0.071** | **0.011** | **0.025** | **0.016** | **0.006** | **0.010** | **0.050** | **0.019** |
| **Significance level** | **0.001** | **0.001** | **0.190** | **0.016** | **0.070** | **0.290** | **0.169** | **0.001** | **0.003** |
| **Groups** |  |  |  |  |  |  |  |  |  |
| **F0Mat, F0Pat** | **0.001** | **0.003** | **0.001** | **0.003** | ns | **0.005** | **0.001** | **0.018** | **0.036** |
| **F0Mat, F0N** | ns | **0.001** | ns | ns | ns | ns | ns | ns | ns |
| **F0Pat, F0N** | **0.002** | **0.001** | ns | **0.046** | **0.041** | ns | ns | ns | **0.036** |
| **F1-bacteria** | **<0.001***** | **<0.001***** | **<0.001***** | ns | **<0.001***** | **<0.001***** | **0.036*** | **0.002**** | **<0.001***** |
| **ANOSIM-Global R** | **0.058** | **0.043** | **0.038** |  | **0.073** | **0.014** | **0.012** | **0.002** | **0.017** |
| **Significance level** | **0.001** | **0.001** | **0.001** |  | **0.001** | **0.001** | **0.001** | **0.268** | **0.004** |
| **Groups** |  |  |  |  |  |  |  |  |  |
| **F1V+, F1T+** | ns | ns | ns |  | ns | ns | ns | ns | ns |
| **F1V+, F1N** | **0.001** | **0.001** | **0.001** |  | **0.001** | **0.009** | **0.007** | **0.008** | **0.005** |
| **F1T+, F1N** | **0.001** | **0.001** | **0.001** |  | **0.001** | **0.005** | **0.008** | **0.004** | **0.001** |
| **F0-bacteria x F1-bacteria** | ns | ns | ns | ns | ns | ns | ns | ns | **0.006**** |
| **ANOSIM-Global R** |  |  |  |  |  |  |  |  | **0.024** |
| **Significance level** |  |  |  |  |  |  |  |  | **0.160** |
| **Groups** |  |  |  |  |  |  |  |  |  |
| **F0V+/F1V+, F0V+/F1T+** |  |  |  |  |  |  |  |  | ns |
| **F0V+/F1V+, F0V+/F1N** |  |  |  |  |  |  |  |  | **0.019** |
| **F0V+/F1V+, F0T+/F1V+** |  |  |  |  |  |  |  |  | ns |
| **F0V+/F1V+, F0T+/F1T+** |  |  |  |  |  |  |  |  | ns |
| **F0V+/F1V+, F0T+/F1N** |  |  |  |  |  |  |  |  | ns |
| **F0V+/F1V+, F0N/F1V+** |  |  |  |  |  |  |  |  | ns |
| **F0V+/F1V+, F0N/F1T+** |  |  |  |  |  |  |  |  | ns |
| **F0V+/F1V+, F0N/F1N** |  |  |  |  |  |  |  |  | ns |
| **F0V+/F1T+, F0V+/F1N** |  |  |  |  |  |  |  |  | **0.01** |
| **F0V+/F1T+, F0T+/F1V+** |  |  |  |  |  |  |  |  | ns |
| **F0V+/F1T+, F0T+/F1T+** |  |  |  |  |  |  |  |  | ns |
| **F0V+/F1T+, F0T+/F1N** |  |  |  |  |  |  |  |  | ns |
| **F0V+/F1T+, F0N/F1V+** |  |  |  |  |  |  |  |  | ns |
| **F0V+/F1T+, F0N/F1T+** |  |  |  |  |  |  |  |  | **0.028** |
| **F0V+/F1T+, F0N/F1N** |  |  |  |  |  |  |  |  | **0.046** |
| **F0V+/F1N, F0T+/F1V+** |  |  |  |  |  |  |  |  | ns |
| **F0V+/F1N, F0T+/F1T+** |  |  |  |  |  |  |  |  | **0.044** |
| **F0V+/F1N, F0T+/F1N** |  |  |  |  |  |  |  |  | ns |
| **F0V+/F1N, F0N/F1V+** |  |  |  |  |  |  |  |  | ns |
| **F0V+/F1N, F0N/F1T+** |  |  |  |  |  |  |  |  | **0.043** |
| **F0V+/F1N, F0N/F1N** |  |  |  |  |  |  |  |  | ns |
| **F0T+/F1V+, F0T+/F1T+** |  |  |  |  |  |  |  |  | ns |
| **F0T+/F1V+, F0T+/F1N** |  |  |  |  |  |  |  |  | ns |
| **F0T+/F1V+, F0N/F1V+** |  |  |  |  |  |  |  |  | ns |
| **F0T+/F1V+, F0N/F1T+** |  |  |  |  |  |  |  |  | ns |
| **F0T+/F1V+, F0N/F1N** |  |  |  |  |  |  |  |  | ns |
| **F0T+/F1T+, F0T+/F1N** |  |  |  |  |  |  |  |  | ns |
| **F0T+/F1T+, F0N/F1V+** |  |  |  |  |  |  |  |  | ns |
| **F0T+/F1T+, F0N/F1T+** |  |  |  |  |  |  |  |  | ns |
| **F0T+/F1T+, F0N/F1N** |  |  |  |  |  |  |  |  | ns |
| **F0T+/F1N, F0N/F1V+** |  |  |  |  |  |  |  |  | ns |
| **F0T+/F1N, F0N/F1T+** |  |  |  |  |  |  |  |  | **0.033** |
| **F0T+/F1N, F0N/F1N** |  |  |  |  |  |  |  |  | ns |
| **F0N/F1V+, F0N/F1T+** |  |  |  |  |  |  |  |  | ns |
| **F0N/F1V+, F0N/F1N** |  |  |  |  |  |  |  |  | ns |
| **F0N/F1T+, F0N/F1N** |  |  |  |  |  |  |  |  | ns |
| **F0-sex x F1-bacteria** | **0.002**** | **0.003**** | ns | ns | **0.004*** | **0.003**** | **0.008**** | **0.005**** | ns |
| **ANOSIM-Global R** | **0.082** | **0.090** |  |  | **0.076** | **0.026** | **0.027** | **0.022** |  |
| **Significance level** | **0.001** | **0.001** |  |  | **0.001** | **0.001** | **0.004** | **0.003** |  |
| **Groups** |  |  |  |  |  |  |  |  |  |
| **F0Mat/F1V+, F0Mat/F1T+** | ns | ns |  |  | ns | ns | ns | ns |  |
| **F0Mat/F1V+, F0Mat/F1N** | **0.001** | **0.001** |  |  | **0.001** | **0.03** | ns | **0.017** |  |
| **F0Mat/F1V+, F0Pat/F1V+** | **0.008** | **0.01** |  |  | ns | **ns** | **0.024** | **ns** |  |
| **F0Mat/F1V+, F0Pat/F1T+** | **0.009** | **0.021** |  |  | ns | **0.004** | **0.003** | **0.002** |  |
| **F0Mat/F1V+, F0Pat/F1N** | **0.001** | **0.001** |  |  | **0.001** | **0.165** | ns | ns |  |
| **F0Mat/F1V+, F0N/F1V+** | ns | **0.005** |  |  | ns | ns | ns | ns |  |
| **F0Mat/F1V+, F0N/F1T+** | ns | **0.015** |  |  | ns | ns | ns | ns |  |
| **F0Mat/F1V+, F0N/F1N** | ns | **0.029** |  |  | **0.002** | ns | ns | ns |  |
| **F0Mat/F1T+, F0Mat/F1N** | **0.001** | **0.001** |  |  | **0.001** | **0.009** | **0.059** | **0.007** |  |
| **F0Mat/F1T+, F0Pat/F1V+** | **0.01** | **0.017** |  |  | **0.026** | **0.042** | **0.003** | **0.031** |  |
| **F0Mat/F1T+, F0Pat/F1T+** | **0.001** | **0.012** |  |  | ns | **0.001** | **0.001** | **0.001** |  |
| **F0Mat/F1T+, F0Pat/F1N** | **0.001** | **0.001** |  |  | **0.001** | **0.028** | **0.089** | **0.038** |  |
| **F0Mat/F1T+, F0N/F1V+** | ns | **0.003** |  |  | ns | ns | ns | ns |  |
| **F0Mat/F1T+, F0N/F1T+** | ns | **0.008** |  |  | **0.015** | ns | ns | ns |  |
| **F0Mat/F1T+, F0N/F1N** | **0.024** | ns |  |  | **0.001** | ns | ns | ns |  |
| **F0Mat/F1N, F0Pat/F1V+** | **0.001** | **0.001** |  |  | **0.004** | **0.025** | **0.037** | ns |  |
| **F0Mat/F1N, F0Pat/F1T+** | **0.001** | **0.001** |  |  | **0.001** | **0.015** | **0.011** | ns |  |
| **F0Mat/F1N, F0Pat/F1N** | ns | ns |  |  | **0.014** | ns | **0.046** | ns |  |
| **F0Mat/F1N, F0N/F1V+** | **0.001** | **0.001** |  |  | **0.013** | ns | ns | ns |  |
| **F0Mat/F1N, F0N/F1T+** | **0.004** | **0.001** |  |  | **0.003** | ns | ns | ns |  |
| **F0Mat/F1N, F0N/F1N** | ns | **0.052** |  |  | ns | ns | ns | ns |  |
| **F0Pat/F1V+, F0Pat/F1T+** | ns | ns |  |  | ns | ns | ns | ns |  |
| **F0Pat/F1V+, F0Pat/F1N** | **0.02** | **0.006** |  |  | ns | **0.056** | **0.013** | ns |  |
| **F0Pat/F1V+, F0N/F1V+** | ns | **0.013** |  |  | ns | **ns** | ns | **0.026** |  |
| **F0Pat/F1V+, F0N/F1T+** | ns | **0.019** |  |  | ns | **ns** | ns | ns |  |
| **F0Pat/F1V+, F0N/F1N** | **0.041** | **0.026** |  |  | ns | **ns** | **0.03** | **ns** |  |
| **F0Pat/F1T+, F0Pat/F1N** | **0.001** | **0.003** |  |  | **0.009** | **0.005** | **0.008** | **0.044** |  |
| **F0Pat/F1T+, F0N/F1V+** | **0.012** | **0.003** |  |  | ns | ns | **ns** | ns |  |
| **F0Pat/F1T+, F0N/F1T+** | **0.011** | **0.004** |  |  | **0.03** | ns | **ns** | ns |  |
| **F0Pat/F1T+, F0N/F1N** | **0.005** | **0.007** |  |  | **0.007** | ns | **0.003** | ns |  |
| **F0Pat/F1N, F0N/F1V+** | **0.001** | **0.001** |  |  | ns | ns | ns | ns |  |
| **F0Pat/F1N, F0N/F1T+** | **0.001** | **0.001** |  |  | **0.035** | ns | ns | ns |  |
| **F0Pat/F1N, F0N/F1N** | ns | ns |  |  | ns | ns | ns | ns |  |
| **F0N/F1V+, F0N/F1T+** | ns | ns |  |  | ns | ns | ns | ns |  |
| **F0N/F1V+, F0N/F1N** | **0.023** | **0.048** |  |  | ns | **0.056** | **0.02** | ns |  |
| **F0N/F1T+, F0N/F1N** | ns | ns |  |  | ns | ns | **0.045** | ns |  |
| **F0-bacteria x F1-bacteria x F0-sex** | ns | ns | ns | **0.044*** | ns | ns | ns | ns | ns |
| **ANOSIM-Global R** |  |  |  | **0.079** |  |  |  |  |  |
| **Significance level** |  |  |  | **0.001** |  |  |  |  |  |
| **Groups** |  |  |  |  |  |  |  |  |  |
| **F0Mat/F0V+/F1V+, F0Mat/F0V+/F1T+** |  |  |  | ns |  |  |  |  |  |
| **F0Mat/F0V+/F1V+, F0Mat/F0V+/F1N** |  |  |  | ns |  |  |  |  |  |
| **F0Mat/F0V+/F1V+, F0Mat/F0T+/F1V+** |  |  |  | **0.001** |  |  |  |  |  |
| **F0Mat/F0V+/F1V+, F0Mat/F0T+/F1T+** |  |  |  | **0.003** |  |  |  |  |  |
| **F0Mat/F0V+/F1V+, F0Mat/F0T+/F1N** |  |  |  | **0.001** |  |  |  |  |  |
| **F0Mat/F0V+/F1V+, F0Pat/F0V+/F1V+** |  |  |  | **0.008** |  |  |  |  |  |
| **F0Mat/F0V+/F1V+, F0Pat/F0V+/F1T+** |  |  |  | **0.023** |  |  |  |  |  |
| **F0Mat/F0V+/F1V+, F0Pat/F0V+/F1N** |  |  |  | ns |  |  |  |  |  |
| **F0Mat/F0V+/F1V+, F0Pat/F0T+/F1V+** |  |  |  | **0.047** |  |  |  |  |  |
| **F0Mat/F0V+/F1V+, F0Pat/F0T+/F1T+** |  |  |  | **0.006** |  |  |  |  |  |
| **F0Mat/F0V+/F1V+, F0Pat/F0T+/F1N** |  |  |  | ns |  |  |  |  |  |
| **F0Mat/F0V+/F1V+, F0N/F1V+** |  |  |  | **0.012** |  |  |  |  |  |
| **F0Mat/F0V+/F1V+, F0N/F1T+** |  |  |  | **0.008** |  |  |  |  |  |
| **F0Mat/F0V+/F1V+, F0N/F1N** |  |  |  | **0.001** |  |  |  |  |  |
| **F0Mat/F0V+/F1T+, F0Mat/F0V+/F1N** |  |  |  | ns |  |  |  |  |  |
| **F0Mat/F0V+/F1T+, F0Mat/F0T+/F1V+** |  |  |  | **0.001** |  |  |  |  |  |
| **F0Mat/F0V+/F1T+, F0Mat/F0T+/F1T+** |  |  |  | **0.008** |  |  |  |  |  |
| **F0Mat/F0V+/F1T+, F0Mat/F0T+/F1N** |  |  |  | **0.001** |  |  |  |  |  |
| **F0Mat/F0V+/F1T+, F0Pat/F0V+/F1V+** |  |  |  | ns |  |  |  |  |  |
| **F0Mat/F0V+/F1T+, F0Pat/F0V+/F1T+** |  |  |  | ns |  |  |  |  |  |
| **F0Mat/F0V+/F1T+, F0Pat/F0V+/F1N** |  |  |  | ns |  |  |  |  |  |
| **F0Mat/F0V+/F1T+, F0Pat/F0T+/F1V+** |  |  |  | **0.017** |  |  |  |  |  |
| **F0Mat/F0V+/F1T+, F0Pat/F0T+/F1T+** |  |  |  | **0.019** |  |  |  |  |  |
| **F0Mat/F0V+/F1T+, F0Pat/F0T+/F1N** |  |  |  | ns |  |  |  |  |  |
| **F0Mat/F0V+/F1T+, F0N/F1V+** |  |  |  | **0.037** |  |  |  |  |  |
| **F0Mat/F0V+/F1T+, F0N/F1T+** |  |  |  | **0.046** |  |  |  |  |  |
| **F0Mat/F0V+/F1T+, F0N/F1N** |  |  |  | **0.026** |  |  |  |  |  |
| **F0Mat/F0V+/F1N, F0Mat/F0T+/F1V+** |  |  |  | **0.001** |  |  |  |  |  |
| **F0Mat/F0V+/F1N, F0Mat/F0T+/F1T+** |  |  |  | **0.038** |  |  |  |  |  |
| **F0Mat/F0V+/F1N, F0Mat/F0T+/F1N** |  |  |  | **0.006** |  |  |  |  |  |
| **F0Mat/F0V+/F1N, F0Pat/F0V+/F1V+** |  |  |  | ns |  |  |  |  |  |
| **F0Mat/F0V+/F1N, F0Pat/F0V+/F1T+** |  |  |  | ns |  |  |  |  |  |
| **F0Mat/F0V+/F1N, F0Pat/F0V+/F1N** |  |  |  | ns |  |  |  |  |  |
| **F0Mat/F0V+/F1N, F0Pat/F0T+/F1V+** |  |  |  | **0.018** |  |  |  |  |  |
| **F0Mat/F0V+/F1N, F0Pat/F0T+/F1T+** |  |  |  | ns |  |  |  |  |  |
| **F0Mat/F0V+/F1N, F0Pat/F0T+/F1N** |  |  |  | ns |  |  |  |  |  |
| **F0Mat/F0V+/F1N, F0N/F1V+** |  |  |  | ns |  |  |  |  |  |
| **F0Mat/F0V+/F1N, F0N/F1T+** |  |  |  | ns |  |  |  |  |  |
| **F0Mat/F0V+/F1N, F0N/F1N** |  |  |  | ns |  |  |  |  |  |
| **F0Mat/F0T+/F1V+, F0Mat/F0T+/F1T+** |  |  |  | ns |  |  |  |  |  |
| **F0Mat/F0T+/F1V+, F0Mat/F0T+/F1N** |  |  |  | ns |  |  |  |  |  |
| **F0Mat/F0T+/F1V+, F0Pat/F0V+/F1V+** |  |  |  | **0.001** |  |  |  |  |  |
| **F0Mat/F0T+/F1V+, F0Pat/F0V+/F1T+** |  |  |  | **0.001** |  |  |  |  |  |
| **F0Mat/F0T+/F1V+, F0Pat/F0V+/F1N** |  |  |  | **0.001** |  |  |  |  |  |
| **F0Mat/F0T+/F1V+, F0Pat/F0T+/F1V+** |  |  |  | **0.006** |  |  |  |  |  |
| **F0Mat/F0T+/F1V+, F0Pat/F0T+/F1T+** |  |  |  | ns |  |  |  |  |  |
| **F0Mat/F0T+/F1V+, F0Pat/F0T+/F1N** |  |  |  | **0.02** |  |  |  |  |  |
| **F0Mat/F0T+/F1V+, F0N/F1V+** |  |  |  | **0.008** |  |  |  |  |  |
| **F0Mat/F0T+/F1V+, F0N/F1T+** |  |  |  | ns |  |  |  |  |  |
| **F0Mat/F0T+/F1V+, F0N/F1N** |  |  |  | **0.001** |  |  |  |  |  |
| **F0Mat/F0T+/F1T+, F0Mat/F0T+/F1N** |  |  |  | ns |  |  |  |  |  |
| **F0Mat/F0T+/F1T+, F0Pat/F0V+/F1V+** |  |  |  | **0.002** |  |  |  |  |  |
| **F0Mat/F0T+/F1T+, F0Pat/F0V+/F1T+** |  |  |  | **0.003** |  |  |  |  |  |
| **F0Mat/F0T+/F1T+, F0Pat/F0V+/F1N** |  |  |  | **0.003** |  |  |  |  |  |
| **F0Mat/F0T+/F1T+, F0Pat/F0T+/F1V+** |  |  |  | **0.013** |  |  |  |  |  |
| **F0Mat/F0T+/F1T+, F0Pat/F0T+/F1T+** |  |  |  | ns |  |  |  |  |  |
| **F0Mat/F0T+/F1T+, F0Pat/F0T+/F1N** |  |  |  | **0.033** |  |  |  |  |  |
| **F0Mat/F0T+/F1T+, F0N/F1V+** |  |  |  | ns |  |  |  |  |  |
| **F0Mat/F0T+/F1T+, F0N/F1T+** |  |  |  | ns |  |  |  |  |  |
| **F0Mat/F0T+/F1T+, F0N/F1N** |  |  |  | ns |  |  |  |  |  |
| **F0Mat/F0T+/F1N, F0Pat/F0V+/F1V+** |  |  |  | **0.002** |  |  |  |  |  |
| **F0Mat/F0T+/F1N, F0Pat/F0V+/F1T+** |  |  |  | **0.002** |  |  |  |  |  |
| **F0Mat/F0T+/F1N, F0Pat/F0V+/F1N** |  |  |  | **0.002** |  |  |  |  |  |
| **F0Mat/F0T+/F1N, F0Pat/F0T+/F1V+** |  |  |  | **0.023** |  |  |  |  |  |
| **F0Mat/F0T+/F1N, F0Pat/F0T+/F1T+** |  |  |  | **0.04** |  |  |  |  |  |
| **F0Mat/F0T+/F1N, F0Pat/F0T+/F1N** |  |  |  | ns |  |  |  |  |  |
| **F0Mat/F0T+/F1N, F0N/F1V+** |  |  |  | **0.017** |  |  |  |  |  |
| **F0Mat/F0T+/F1N, F0N/F1T+** |  |  |  | **0.022** |  |  |  |  |  |
| **F0Mat/F0T+/F1N, F0N/F1N** |  |  |  | **0.002** |  |  |  |  |  |
| **F0Pat/F0V+/F1V+, F0Pat/F0V+/F1T+** |  |  |  | ns |  |  |  |  |  |
| **F0Pat/F0V+/F1V+, F0Pat/F0V+/F1N** |  |  |  | ns |  |  |  |  |  |
| **F0Pat/F0V+/F1V+, F0Pat/F0T+/F1V+** |  |  |  | **0.001** |  |  |  |  |  |
| **F0Pat/F0V+/F1V+, F0Pat/F0T+/F1T+** |  |  |  | **0.044** |  |  |  |  |  |
| **F0Pat/F0V+/F1V+, F0Pat/F0T+/F1N** |  |  |  | **0.016** |  |  |  |  |  |
| **F0Pat/F0V+/F1V+, F0N/F1V+** |  |  |  | **0.029** |  |  |  |  |  |
| **F0Pat/F0V+/F1V+, F0N/F1T+** |  |  |  | **0.004** |  |  |  |  |  |
| **F0Pat/F0V+/F1V+, F0N/F1N** |  |  |  | **0.025** |  |  |  |  |  |
| **F0Pat/F0V+/F1T+, F0Pat/F0V+/F1N** |  |  |  | ns |  |  |  |  |  |
| **F0Pat/F0V+/F1T+, F0Pat/F0T+/F1V+** |  |  |  | **0.004** |  |  |  |  |  |
| **F0Pat/F0V+/F1T+, F0Pat/F0T+/F1T+** |  |  |  | ns |  |  |  |  |  |
| **F0Pat/F0V+/F1T+, F0Pat/F0T+/F1N** |  |  |  | **0.032** |  |  |  |  |  |
| **F0Pat/F0V+/F1T+, F0N/F1V+** |  |  |  | **0.026** |  |  |  |  |  |
| **F0Pat/F0V+/F1T+, F0N/F1T+** |  |  |  | **0.024** |  |  |  |  |  |
| **F0Pat/F0V+/F1T+, F0N/F1N** |  |  |  | **0.045** |  |  |  |  |  |
| **F0Pat/F0V+/F1N, F0Pat/F0T+/F1V+** |  |  |  | **0.002** |  |  |  |  |  |
| **F0Pat/F0V+/F1N, F0Pat/F0T+/F1T+** |  |  |  | **0.025** |  |  |  |  |  |
| **F0Pat/F0V+/F1N, F0Pat/F0T+/F1N** |  |  |  | ns |  |  |  |  |  |
| **F0Pat/F0V+/F1N, F0N/F1V+** |  |  |  | **0.04** |  |  |  |  |  |
| **F0Pat/F0V+/F1N, F0N/F1T+** |  |  |  | **0.001** |  |  |  |  |  |
| **F0Pat/F0V+/F1N, F0N/F1N** |  |  |  | **0.031** |  |  |  |  |  |
| **F0Pat/F0T+/F1V+, F0Pat/F0T+/F1T+** |  |  |  | ns |  |  |  |  |  |
| **F0Pat/F0T+/F1V+, F0Pat/F0T+/F1N** |  |  |  | ns |  |  |  |  |  |
| **F0Pat/F0T+/F1V+, F0N/F1V+** |  |  |  | **0.006** |  |  |  |  |  |
| **F0Pat/F0T+/F1V+, F0N/F1T+** |  |  |  | ns |  |  |  |  |  |
| **F0Pat/F0T+/F1V+, F0N/F1N** |  |  |  | **0.001** |  |  |  |  |  |
| **F0Pat/F0T+/F1T+, F0Pat/F0T+/F1N** |  |  |  | ns |  |  |  |  |  |
| **F0Pat/F0T+/F1T+, F0N/F1V+** |  |  |  | ns |  |  |  |  |  |
| **F0Pat/F0T+/F1T+, F0N/F1T+** |  |  |  | ns |  |  |  |  |  |
| **F0Pat/F0T+/F1T+, F0N/F1N** |  |  |  | **0.02** |  |  |  |  |  |
| **F0Pat/F0T+/F1N, F0N/F1V+** |  |  |  | ns |  |  |  |  |  |
| **F0Pat/F0T+/F1N, F0N/F1T+** |  |  |  | **0.046** |  |  |  |  |  |
| **F0Pat/F0T+/F1N, F0N/F1N** |  |  |  | **0.003** |  |  |  |  |  |
| **F0N/F1V+, F0N/F1T+** |  |  |  | ns |  |  |  |  |  |
| **F0N/F1V+, F0N/F1N** |  |  |  | ns |  |  |  |  |  |
| **F0N/F1T+, F0N/F1N** |  |  |  | ns |  |  |  |  |  |

**Table S2: Results from PERMANOVA and ANOSIM analysis of four-month-old per functional gene categories and immune cell measurements.** Multivariate ANOSIM was performed following significant PERMANOVA to assess differences in the gene expression profiles and immune cell measurement per treatment groups applying pairwise comparison based on a Bray-Curtis distance matrix and 999 permutations. Pairwise comparison was conducted for following fixed factors and their interaction: F0-bacteria treatment effect (parental control (F0N), parental *Vibrio* (F0V+) vs *Tenacibaculum* (F0T+)), F0-sex´ (maternal (F0Mat), paternal (F0Pat) vs control (F0N)) and `F1-bacteria´ (F1-offspring control (F1N), F1-offspring *Vibrio* (F1V+) and *Tenacibaculum* (F1T+)).

| **4-month-old** | **Immune genes (29)** | **Innate genes (13)** | **Innate & Adaptive genes (5)** | **Adaptive genes (8)** | **Complemet component genes (3)** | **Epigenetic genes (15)** | **DNAmethylation genes (5)** | **Histone de/methylation genes (4)** | **Histone de/acetylation**  **genes (5)** | **Cell counts** | **Cell counts blood** | **Cell counts hk** |
| --- | --- | --- | --- | --- | --- | --- | --- | --- | --- | --- | --- | --- |
| **F0-bacteria** | **0.002**** | **0.001***** | ns | ns | ns | ns | **0.020*** | ns | ns | **0.001***** | **0.006**** | **0.001***** |
| **Global R** | 0.061 | 0.042 |  |  |  |  | 0.088 |  |  | 0.162 | 0.168 | 0.159 |
| **Significance level** | 0.001 | 0.005 |  |  |  |  | 0.001 |  |  | 0.001 | 0.001 | 0.001 |
| **Groups** |  |  |  |  |  |  |  |  |  |  |  |  |
| **F0V+, F0T+** | **0.004** | **0.003** |  |  |  |  | **0.002** |  |  | **0.001** | **0.001** | **0.007** |
| **F0V+, F0N** | **0.001** | **0.005** |  |  |  |  | **0.001** |  |  | **0.001** | **0.003** | **0.001** |
| **F0T+, F0N** | ns | ns |  |  |  |  | ns |  |  | **0.001** | **0.001** | **0.001** |
| **F0-sex** | **0.002**** | **0.001***** | ns | ns | ns | ns | **0.020*** | ns | ns | **0.001***** | **0.006**** | **0.004**** |
| **ANOSIM-Global R** | 0.052 | 0.035 |  |  |  |  | 0.020 |  |  | 0.129 | 0.111 | 0.139 |
| **Significance level** | 0.001 | 0.04 |  |  |  |  | 0.046 |  |  | 0.001 | 0.001 | 0.001 |
| **Groups** |  |  |  |  |  |  |  |  |  |  |  |  |
| **F0Mat, F0Pat** | **0.009** | **0.019** |  |  |  |  | ns |  |  | ns | ns | ns |
| **F0Mat, F0N** | **0.005** | **ns** |  |  |  |  | **0.003** |  |  | **0.001** | **0.001** | **0.001** |
| **F0Pat, F0N** | **0.001** | **0.003** |  |  |  |  | **0.003** |  |  | **0.001** | **0.003** | **0.001** |
| **F1-bacteria** | **<0.001***** | **<0.001***** | **<0.001***** | ns | ns | ns | **ns** | ns | ns | **<0.001***** | **<0.001***** | **<0.001***** |
| **ANOSIM-Global R** | 0.059 | 0.08 | 0.032 |  |  |  | ns |  |  | 0.036 | 0.008 | 0.06 |
| **Significance level** | 0.001 | 0.001 | 0.001 |  |  |  | ns |  |  | 0.006 | 0.004 | 0.006 |
| **Groups** |  |  |  |  |  |  |  |  |  |  |  |  |
| **F1V+, F1T+** | **ns** | **ns** | **ns** |  |  |  | ns |  |  | **ns** | **ns** | **ns** |
| **F1V+, F1N** | **0.001** | **0.0001** | **0.003** |  |  |  | ns |  |  | **0.002** | **0.005** | **0.002** |
| **F1T+, F1N** | **0.002** | **0.0002** | **0.003** |  |  |  | ns |  |  | **0.004** | **0.004** | **0.001** |
| **F0-bacteria x F1-bacteria** | ns | ns | ns | ns | ns | ns | ns | ns | ns | ns | ns | ns |
| **F0-sex x F1-bacteria** | ns | ns | ns | ns | ns | ns | ns | ns | ns | ns | ns | ns |
| **F0-bacteria x F1-bacteria x F0-sex** | ns | ns | ns | **0.044*** | ns | ns | ns | ns | ns | **0.042*** | ns | **0.008**** |
| **ANOSIM-Global R** |  |  |  | 0.142 |  |  |  |  |  | 0.133 |  | 0.181 |
| **Significance level** |  |  |  | 0.001 |  |  |  |  |  | 0.001 |  | 0.001 |
| **Groups** |  |  |  |  |  |  |  |  |  |  |  |  |
| **F0Mat/F0V+/F1V+, F0Mat/F0V+/F1T+** |  |  |  | ns |  |  |  |  |  | ns |  | ns |
| **F0Mat/F0V+/F1V+, F0Mat/F0V+/F1N** |  |  |  | ns |  |  |  |  |  | ns |  | ns |
| **F0Mat/F0V+/F1V+, F0Mat/F0T+/F1V+** |  |  |  | **0.01** |  |  |  |  |  | **0.037** |  | ns |
| **F0Mat/F0V+/F1V+, F0Mat/F0T+/F1T+** |  |  |  | **0.01** |  |  |  |  |  | ns |  | ns |
| **F0Mat/F0V+/F1V+, F0Mat/F0T+/F1N** |  |  |  | ns |  |  |  |  |  | ns |  | ns |
| **F0Mat/F0V+/F1V+, F0Pat/F0V+/F1V+** |  |  |  | ns |  |  |  |  |  | ns |  | ns |
| **F0Mat/F0V+/F1V+, F0Pat/F0V+/F1T+** |  |  |  | ns |  |  |  |  |  | ns |  | **0.009** |
| **F0Mat/F0V+/F1V+, F0Pat/F0V+/F1N** |  |  |  | ns |  |  |  |  |  | ns |  | ns |
| **F0Mat/F0V+/F1V+, F0Pat/F0T+/F1V+** |  |  |  | ns |  |  |  |  |  | ns |  | ns |
| **F0Mat/F0V+/F1V+, F0Pat/F0T+/F1T+** |  |  |  | ns |  |  |  |  |  | ns |  | ns |
| **F0Mat/F0V+/F1V+, F0Pat/F0T+/F1N** |  |  |  | ns |  |  |  |  |  | ns |  | ns |
| **F0Mat/F0V+/F1V+, F0N/F1V+** |  |  |  | ns |  |  |  |  |  | ns |  | **0.041** |
| **F0Mat/F0V+/F1V+, F0N/F1T+** |  |  |  | ns |  |  |  |  |  | ns |  | **0.006** |
| **F0Mat/F0V+/F1V+, F0N/F1N** |  |  |  | **0.04** |  |  |  |  |  | ns |  | **0.001** |
| **F0Mat/F0V+/F1T+, F0Mat/F0V+/F1N** |  |  |  | **ns** |  |  |  |  |  | ns |  | ns |
| **F0Mat/F0V+/F1T+, F0Mat/F0T+/F1V+** |  |  |  | ns |  |  |  |  |  | **0.048** |  | ns |
| **F0Mat/F0V+/F1T+, F0Mat/F0T+/F1T+** |  |  |  | **0** |  |  |  |  |  | ns |  | ns |
| **F0Mat/F0V+/F1T+, F0Mat/F0T+/F1N** |  |  |  | ns |  |  |  |  |  | ns |  | ns |
| **F0Mat/F0V+/F1T+, F0Pat/F0V+/F1V+** |  |  |  | ns |  |  |  |  |  | ns |  | ns |
| **F0Mat/F0V+/F1T+, F0Pat/F0V+/F1T+** |  |  |  | ns |  |  |  |  |  | ns |  | **0.022** |
| **F0Mat/F0V+/F1T+, F0Pat/F0V+/F1N** |  |  |  | ns |  |  |  |  |  | ns |  | **0.048** |
| **F0Mat/F0V+/F1T+, F0Pat/F0T+/F1V+** |  |  |  | ns |  |  |  |  |  | ns |  | ns |
| **F0Mat/F0V+/F1T+, F0Pat/F0T+/F1T+** |  |  |  | ns |  |  |  |  |  | ns |  | ns |
| **F0Mat/F0V+/F1T+, F0Pat/F0T+/F1N** |  |  |  | ns |  |  |  |  |  | ns |  | ns |
| **F0Mat/F0V+/F1T+, F0N/F1V+** |  |  |  | ns |  |  |  |  |  | ns |  | ns |
| **F0Mat/F0V+/F1T+, F0N/F1T+** |  |  |  | **0.01** |  |  |  |  |  | ns |  | **0.032** |
| **F0Mat/F0V+/F1T+, F0N/F1N** |  |  |  | **0.01** |  |  |  |  |  | ns |  | **0.001** |
| **F0Mat/F0V+/F1N, F0Mat/F0T+/F1V+** |  |  |  | **0** |  |  |  |  |  | **0.037** |  | ns |
| **F0Mat/F0V+/F1N, F0Mat/F0T+/F1T+** |  |  |  | **0** |  |  |  |  |  | ns |  | ns |
| **F0Mat/F0V+/F1N, F0Mat/F0T+/F1N** |  |  |  | **0.02** |  |  |  |  |  | ns |  | ns |
| **F0Mat/F0V+/F1N, F0Pat/F0V+/F1V+** |  |  |  | **0.01** |  |  |  |  |  | ns |  | ns |
| **F0Mat/F0V+/F1N, F0Pat/F0V+/F1T+** |  |  |  | **0** |  |  |  |  |  | **0.026** |  | **0.017** |
| **F0Mat/F0V+/F1N, F0Pat/F0V+/F1N** |  |  |  | ns |  |  |  |  |  | ns |  | ns |
| **F0Mat/F0V+/F1N, F0Pat/F0T+/F1V+** |  |  |  | **0.01** |  |  |  |  |  | **0.043** |  | ns |
| **F0Mat/F0V+/F1N, F0Pat/F0T+/F1T+** |  |  |  | **0.04** |  |  |  |  |  | ns |  | ns |
| **F0Mat/F0V+/F1N, F0Pat/F0T+/F1N** |  |  |  | ns |  |  |  |  |  | ns |  | ns |
| **F0Mat/F0V+/F1N, F0N/F1V+** |  |  |  | **0.01** |  |  |  |  |  | ns |  | ns |
| **F0Mat/F0V+/F1N, F0N/F1T+** |  |  |  | **0** |  |  |  |  |  | ns |  | ns |
| **F0Mat/F0V+/F1N, F0N/F1N** |  |  |  | **0** |  |  |  |  |  | ns |  | **0.007** |
| **F0Mat/F0T+/F1V+, F0Mat/F0T+/F1T+** |  |  |  | ns |  |  |  |  |  | ns |  | ns |
| **F0Mat/F0T+/F1V+, F0Mat/F0T+/F1N** |  |  |  | ns |  |  |  |  |  | ns |  | ns |
| **F0Mat/F0T+/F1V+, F0Pat/F0V+/F1V+** |  |  |  | ns |  |  |  |  |  | ns |  | ns |
| **F0Mat/F0T+/F1V+, F0Pat/F0V+/F1T+** |  |  |  | ns |  |  |  |  |  | ns |  | ns |
| **F0Mat/F0T+/F1V+, F0Pat/F0V+/F1N** |  |  |  | ns |  |  |  |  |  | **0.015** |  | **0.028** |
| **F0Mat/F0T+/F1V+, F0Pat/F0T+/F1V+** |  |  |  | ns |  |  |  |  |  | ns |  | ns |
| **F0Mat/F0T+/F1V+, F0Pat/F0T+/F1T+** |  |  |  | ns |  |  |  |  |  | ns |  | ns |
| **F0Mat/F0T+/F1V+, F0Pat/F0T+/F1N** |  |  |  | **0.03** |  |  |  |  |  | ns |  | ns |
| **F0Mat/F0T+/F1V+, F0N/F1V+** |  |  |  | ns |  |  |  |  |  | ns |  | **0.033** |
| **F0Mat/F0T+/F1V+, F0N/F1T+** |  |  |  | ns |  |  |  |  |  | **0.041** |  | **0.011** |
| **F0Mat/F0T+/F1V+, F0N/F1N** |  |  |  | **0.01** |  |  |  |  |  | **0.002** |  | **0.001** |
| **F0Mat/F0T+/F1T+, F0Mat/F0T+/F1N** |  |  |  | ns |  |  |  |  |  | ns |  | ns |
| **F0Mat/F0T+/F1T+, F0Pat/F0V+/F1V+** |  |  |  | **0.04** |  |  |  |  |  | ns |  | ns |
| **F0Mat/F0T+/F1T+, F0Pat/F0V+/F1T+** |  |  |  | ns |  |  |  |  |  | **0.032** |  | ns |
| **F0Mat/F0T+/F1T+, F0Pat/F0V+/F1N** |  |  |  | ns |  |  |  |  |  | **0.006** |  | **0.009** |
| **F0Mat/F0T+/F1T+, F0Pat/F0T+/F1V+** |  |  |  | ns |  |  |  |  |  | ns |  | ns |
| **F0Mat/F0T+/F1T+, F0Pat/F0T+/F1T+** |  |  |  | ns |  |  |  |  |  | ns |  | ns |
| **F0Mat/F0T+/F1T+, F0Pat/F0T+/F1N** |  |  |  | **0.02** |  |  |  |  |  | ns |  | ns |
| **F0Mat/F0T+/F1T+, F0N/F1V+** |  |  |  | ns |  |  |  |  |  | ns |  | ns |
| **F0Mat/F0T+/F1T+, F0N/F1T+** |  |  |  | ns |  |  |  |  |  | ns |  | **0.033** |
| **F0Mat/F0T+/F1T+, F0N/F1N** |  |  |  | ns |  |  |  |  |  | **0.018** |  | **0.001** |
| **F0Mat/F0T+/F1N, F0Pat/F0V+/F1V+** |  |  |  | ns |  |  |  |  |  | **0.043** |  | **0.009** |
| **F0Mat/F0T+/F1N, F0Pat/F0V+/F1T+** |  |  |  | ns |  |  |  |  |  | **0.026** |  | **0.002** |
| **F0Mat/F0T+/F1N, F0Pat/F0V+/F1N** |  |  |  | ns |  |  |  |  |  | ns |  | ns |
| **F0Mat/F0T+/F1N, F0Pat/F0T+/F1V+** |  |  |  | ns |  |  |  |  |  | ns |  | ns |
| **F0Mat/F0T+/F1N, F0Pat/F0T+/F1T+** |  |  |  | ns |  |  |  |  |  | ns |  | ns |
| **F0Mat/F0T+/F1N, F0Pat/F0T+/F1N** |  |  |  | ns |  |  |  |  |  | ns |  | ns |
| **F0Mat/F0T+/F1N, F0N/F1V+** |  |  |  | ns |  |  |  |  |  | ns |  | ns |
| **F0Mat/F0T+/F1N, F0N/F1T+** |  |  |  | ns |  |  |  |  |  | **0.048** |  | ns |
| **F0Mat/F0T+/F1N, F0N/F1N** |  |  |  | ns |  |  |  |  |  | ns |  | **0.008** |
| **F0Pat/F0V+/F1V+, F0Pat/F0V+/F1T+** |  |  |  | ns |  |  |  |  |  | ns |  | **0.035** |
| **F0Pat/F0V+/F1V+, F0Pat/F0V+/F1N** |  |  |  | ns |  |  |  |  |  | ns |  | **0.013** |
| **F0Pat/F0V+/F1V+, F0Pat/F0T+/F1V+** |  |  |  | ns |  |  |  |  |  | **0.009** |  | **0.022** |
| **F0Pat/F0V+/F1V+, F0Pat/F0T+/F1T+** |  |  |  | ns |  |  |  |  |  | ns |  | ns |
| **F0Pat/F0V+/F1V+, F0Pat/F0T+/F1N** |  |  |  | ns |  |  |  |  |  | **0.045** |  | ns |
| **F0Pat/F0V+/F1V+, F0N/F1V+** |  |  |  | ns |  |  |  |  |  | **0.035** |  | **0.005** |
| **F0Pat/F0V+/F1V+, F0N/F1T+** |  |  |  | **0** |  |  |  |  |  | **0.018** |  | **0.003** |
| **F0Pat/F0V+/F1V+, F0N/F1N** |  |  |  | **0** |  |  |  |  |  | **0.015** |  | **0.001** |
| **F0Pat/F0V+/F1T+, F0Pat/F0V+/F1N** |  |  |  | ns |  |  |  |  |  | **0.017** |  | **0.006** |
| **F0Pat/F0V+/F1T+, F0Pat/F0T+/F1V+** |  |  |  | ns |  |  |  |  |  | **0.004** |  | **0.011** |
| **F0Pat/F0V+/F1T+, F0Pat/F0T+/F1T+** |  |  |  | ns |  |  |  |  |  | **0.037** |  | ns |
| **F0Pat/F0V+/F1T+, F0Pat/F0T+/F1N** |  |  |  | ns |  |  |  |  |  | ns |  | ns |
| **F0Pat/F0V+/F1T+, F0N/F1V+** |  |  |  | ns |  |  |  |  |  | ns |  | ns |
| **F0Pat/F0V+/F1T+, F0N/F1T+** |  |  |  | **0.03** |  |  |  |  |  | ns |  | **0.012** |
| **F0Pat/F0V+/F1T+, F0N/F1N** |  |  |  | **0.01** |  |  |  |  |  | **0.005** |  | **0.001** |
| **F0Pat/F0V+/F1N, F0Pat/F0T+/F1V+** |  |  |  | ns |  |  |  |  |  | **0.024** |  | **0.009** |
| **F0Pat/F0V+/F1N, F0Pat/F0T+/F1T+** |  |  |  | ns |  |  |  |  |  | **0.002** |  | **0.009** |
| **F0Pat/F0V+/F1N, F0Pat/F0T+/F1N** |  |  |  | ns |  |  |  |  |  | **0.024** |  | **0.015** |
| **F0Pat/F0V+/F1N, F0N/F1V+** |  |  |  | ns |  |  |  |  |  | ns |  | ns |
| **F0Pat/F0V+/F1N, F0N/F1T+** |  |  |  | **0.001** |  |  |  |  |  | ns |  | ns |
| **F0Pat/F0V+/F1N, F0N/F1N** |  |  |  | **0.001** |  |  |  |  |  | ns |  | ns |
| **F0Pat/F0T+/F1V+, F0Pat/F0T+/F1T+** |  |  |  | ns |  |  |  |  |  | ns |  | ns |
| **F0Pat/F0T+/F1V+, F0Pat/F0T+/F1N** |  |  |  | ns |  |  |  |  |  | ns |  | ns |
| **F0Pat/F0T+/F1V+, F0N/F1V+** |  |  |  | ns |  |  |  |  |  | ns |  | ns |
| **F0Pat/F0T+/F1V+, F0N/F1T+** |  |  |  | ns |  |  |  |  |  | ns |  | ns |
| **F0Pat/F0T+/F1V+, F0N/F1N** |  |  |  | **0.05** |  |  |  |  |  | ns |  | **0.002** |
| **F0Pat/F0T+/F1T+, F0Pat/F0T+/F1N** |  |  |  | ns |  |  |  |  |  | ns |  | ns |
| **F0Pat/F0T+/F1T+, F0N/F1V+** |  |  |  | ns |  |  |  |  |  | ns |  | ns |
| **F0Pat/F0T+/F1T+, F0N/F1T+** |  |  |  | ns |  |  |  |  |  | ns |  | **0.048** |
| **F0Pat/F0T+/F1T+, F0N/F1N** |  |  |  | **0.02** |  |  |  |  |  | **0.024** |  | **0.001** |
| **F0Pat/F0T+/F1N, F0N/F1V+** |  |  |  | **0.03** |  |  |  |  |  | ns |  | ns |
| **F0Pat/F0T+/F1N, F0N/F1T+** |  |  |  | **0.01** |  |  |  |  |  | ns |  | ns |
| **F0Pat/F0T+/F1N, F0N/F1N** |  |  |  | **0.001** |  |  |  |  |  | **0.042** |  | **0.001** |
| **F0N/F1V+, F0N/F1T+** |  |  |  | ns |  |  |  |  |  | ns |  | ns |
| **F0N/F1V+, F0N/F1N** |  |  |  | **0.01** |  |  |  |  |  | **0.023** |  | **0.005** |
| **F0N/F1T+, F0N/F1N** |  |  |  | ns |  |  |  |  |  | **0.016** |  | **0.008** |

**Table S3: Immune gene contribution (29) of one-week-old juveniles based on the scores of two extracted principle coordinates**. Variance summaries are listed for the two principle components of the Between Class Analysis (BCA) (Inertia %) and contributions of each gene (gene contribution %) on the respective principle components (Axis1 and Axis2). Genes with a contribution of above 25 % summed average contribution, were considered as important genes which added the highest variance to the dimensional space and are marked in bold letters.

|  | **F0-bacteria** | | **F1-bacteria** | | **F0 x F1-bacteria** | | |
| --- | --- | --- | --- | --- | --- | --- | --- |
|  | **Axis 1** | **Axis 2** | **Axis 1** | **Axis 2** | **Axis 1** | **Axis 2** |  |
| Inertia (Variance) % | 66.4 | 33.6 | 93.9 | 6.1 | 47.0 | 23.8 |  |
| *Gene Contribution %* | Contribution % | | Contribution % | | Contribution % | |  |
| *Lymphocyte antigen 75* | 2.9 | 2.5 | 0.8 | 1.6 | 2.6 | 0.9 |  |
| *HIVEP2* | 3.4 | 0.6 | 0.0 | 0.0 | 0.9 | 3.7 |  |
| *HIVEP3* | **5.8** | 0.0 | 0.1 | 0.1 | 1.3 | **6.3** |  |
| *CD45* | **5.5** | 1.4 | 1.1 | 0.6 | 0.3 | **8.9** |  |
| *Integrin* | 0.0 | 2.8 | 5.3 | 0.8 | 2.3 | 4.0 |  |
| *Immunoglobulin light chain* | **12.1** | 0.1 | 0.0 | 0.0 | 3.3 | **8.8** |  |
| *Lymphocyte cytosolic protein 2* | 0.7 | 4.2 | 0.1 | 2.9 | 0.6 | 1.0 |  |
| *Tapasin* | **4.7** | 2.3 | 0.1 | 2.2 | 1.2 | **5.2** |  |
| *Lectin protein I* | **15.4** | 1.7 | 0.8 | 2.5 | 1.9 | **14.8** |  |
| *Lectin protein II* | 2.1 | **11.1** | 0.5 | **4.6** | 0.0 | 2.6 |  |
| *Coagulation factor II* | 0.4 | **19.0** | 0.1 | **5.1** | 0.0 | 0.9 |  |
| *Heat shock protein 60 (Hsp60)* | 0.4 | 0.2 | 0.5 | 0.0 | 0.5 | 0.0 |  |
| *Peptidoglycan recognition protein* | 0.0 | 0.1 | 0.1 | **13.4** | 0.0 | 0.5 |  |
| *Kinesin* | 0.2 | 0.9 | 0.1 | 0.8 | 0.3 | 0.1 |  |
| *Nramp* | 0.0 | 4.2 | 1.2 | **3.7** | 0.3 | 1.5 |  |
| *Allograft inflammation factor* | **7.4** | 3.1 | **27.3** | 1.1 | **26.0** | 2.6 |  |
| *Translocator protein* | 0.1 | 0.5 | **6.0** | **11.4** | 1.8 | **7.2** |  |
| *Transferrin* | 0.5 | 0.3 | 1.0 | 0.7 | 1.1 | 0.0 |  |
| *Calreticulin* | 0.8 | 0.2 | 0.0 | **9.0** | 0.1 | 0.0 |  |
| *Interferon* | 3.9 | 1.9 | **14.8** | 0.1 | **13.7** | 1.7 |  |
| *Interleukin 8* | 0.4 | **18.1** | 0.8 | 2.9 | 0.1 | 1.8 |  |
| *Interleukin 10* | 2.0 | 0.2 | **13.3** | 0.0 | **10.4** | 2.4 |  |
| *LPS induced TNFα factor* | 0.1 | **6.0** | 1.5 | 0.7 | 0.7 | 1.0 |  |
| *Tyroproteinkinase* | 0.9 | 0.2 | 1.0 | 0.0 | 1.5 | 0.1 |  |
| *Chemokine 7* | **17.6** | 0.3 | 0.5 | 0.3 | 3.4 | **18.4** |  |
| *Ik-cytokine* | 0.1 | **9.5** | 0.3 | **10.6** | 0.0 | 1.8 |  |
| *Complement component 3* | **5.9** | 0.1 | **18.0** | **7.5** | **17.9** | 1.4 |  |
| *Complement component 1* | **6.7** | **4.8** | 3.3 | **3.7** | **7.2** | 0.8 |  |
| *Complement component 9* | 0.1 | 3.8 | 1.7 | **13.9** | 0.7 | 1.8 |  |

**Table S4: Immune gene contribution (29) of four-month-old juveniles based on the scores of two extracted principle coordinates.** Variance summaries are listed for the two principle components of the Between Class Analysis (BCA) (Inertia %) and contributions of each gene (gene contribution %) on the respective principle components (Axis1 and Axis2). Genes with a contribution of above 25 % summed average contribution, were considered as important genes which added the highest variance to the dimensional space and are marked in bold letters.

|  | **F0-bacteria** | | **F1-bacteria** | | **F0 x F1-bacteria** | |
| --- | --- | --- | --- | --- | --- | --- |
|  | **Axis 1** | **Axis 2** | **Axis 1** | **Axis 2** | **Axis 1** | **Axis 2** |
| Inertia (Variance) % | 89.2 | 10.8 | 87.5 | 12.5 | 34.6 | 28.6 |
| *Gene Contribution %* | Contribution % | | Contribution % | | Contribution % | |
| *Lymphocyte antigen 75* | 0.7 | 3.2 | 5.0 | 0.1 | 2.1 | 3.33 |
| *HIVEP2* | **7.7** | 2.5 | 0.5 | **9.5** | **5.99** | 3.35 |
| *HIVEP3* | **10.0** | 1.0 | 2.4 | **9.7** | **6.41** | **8.15** |
| *CD45* | 0.0 | 0.0 | 1.7 | 0.2 | 0.17 | 1.13 |
| *Integrin* | 2.1 | 0.6 | 2.4 | 1.5 | 1.03 | 3.43 |
| *Immunoglobulin light chain* | **2.9** | **7.6** | 0.1 | 1.1 | 2.62 | 0.43 |
| *Lymphocyte cytosolic protein 2* | 1.0 | 0.5 | 1.8 | 0.3 | 0.47 | 3.01 |
| *Tapasin* | 0.5 | 0.2 | 0.2 | 2.3 | 0.57 | 0.36 |
| *Lectin protein I* | 0.1 | 1.7 | 0.8 | 2.0 | 0.08 | 0.49 |
| *Lectin protein II* | 0.4 | 4.0 | 1.7 | 3.8 | 0.85 | 0.43 |
| *Coagulation factor II* | 1.9 | 4.1 | 1.7 | **7.7** | 0.25 | 3.56 |
| *Heat shock protein 60 (Hsp60)* | **6.2** | 3.2 | 3.2 | 5.0 | **7.11** | 0.41 |
| *Peptidoglycan recognition protein* | **7.3** | 0.7 | 0.8 | 1.7 | **6.36** | 0.16 |
| *Kinesin* | **5.7** | 4.6 | 1.5 | 1.1 | **6.07** | 0.03 |
| *Nramp* | **4.0** | 3.4 | 0.2 | 0.1 | **3.67** | 0.16 |
| *Allograft inflammmation factor* | 2.6 | 0.2 | **12.4** | 3.3 | **8.21** | **7.19** |
| *Translocator protein* | 0.2 | **27.2** | 1.1 | 1.6 | 0.01 | 0.56 |
| *Transferrin* | 0.7 | **7.6** | **16.3** | 2.8 | **4.8** | **13.29** |
| *Calreticulin* | 0.1 | **7.5** | 2.2 | 0.2 | 0.81 | 1.5 |
| *Interferon* | 1.3 | 2.1 | **27.9** | 0.2 | **7.37** | **21.34** |
| *Interleukin 8* | **4.7** | 0.1 | 0.3 | **6.0** | 0.52 | 0.28 |
| *Interleukin 10* | 0.6 | 4.1 | 0.0 | 3.9 | **4.53** | 0.49 |
| *LPS induced TNFα factor* | 0.3 | 1.1 | 2.4 | **27.2** | 0.11 | 3.16 |
| *Tyroproteinkinase* | **10.9** | 0.5 | 0.0 | 0.5 | **7.97** | 1.8 |
| *Chemokine 7* | 2.4 | 0.2 | **9.5** | **7.3** | 0.26 | **13.09** |
| *Ik-cytokine* | 2.5 | 0.0 | 0.5 | 0.1 | 2.34 | 1.8 |
| *Complement component 3* | **13.0** | 3.2 | 1.6 | 0.9 | **7.05** | 0.1 |
| *Complement component 1* | **5.6** | **7.9** | 0.1 | 0.1 | **9.81** | 1.92 |
| *Complement component 9* | **4.8** | 1.3 | 2.0 | 0.0 | 2.45 | 5.07 |

**Table S5: Epigenetic gene contribution (15) of one-week-old juveniles based on the scores of two extracted principle coordinates.** Variance summaries are listed for the two principle components of the Between Class Analysis (BCA) (Inertia %) and contributions of each gene (gene contribution %) on the respective principle components (Axis1 and Axis2). Genes with a contribution of above 25 % summed average contribution, were considered as important genes which added the highest variance to the dimensional space and are marked in bold letters.

|  | **F0-bacteria** | | **F1-bacteria** | |
| --- | --- | --- | --- | --- |
|  | **Axis 1** | **Axis 2** | **Axis 1** | **Axis 2** |
| Inertia (Variance) % | 87.7 | 12.3 | 83.8 | 16.2 |
| *Gene Contribution %* | Contribution % | | Contribution % | |
| *Transcription factor 8* | **11.6** | 1.8 | 0.1 | **11.2** |
| *DNA-methyltransferase 1* | 0.2 | 2.2 | **9.3** | 0.2 |
| *DNA-methyltransferase 3a* | **7.5** | 0.5 | 6.2 | 2.3 |
| *DNA-methyltransferase 3b* | **10.7** | 0.0 | 0.1 | 1.3 |
| *HemK2-methyltransferase* | 5.6 | **10.4** | 6.0 | 2.3 |
| *N6admet- methyltransferase* | 3.1 | 1.2 | 0.1 | 5.5 |
| *Lysine specific demethylase 5B (JmjcPhD)* | 1.9 | **21.8** | **12.7** | 0.1 |
| *Lysine specific demethylase (No66)* | **6.9** | **16.0** | 0.3 | **19.9** |
| *Lysine specific demethylase 6A (TPR)* | 0.6 | **7.5** | 8.0 | 0.2 |
| *Histone methyltransferase (ASH2)* | **11.9** | **21.9** | 6.7 | 6.0 |
| *Histone acetyltransferase KAT2A (BROMO)* | **24.6** | 1.7 | 0.0 | **13.4** |
| *Histone acetyltransferase HAT1 (MYST)* | **7.7** | 0.0 | 6.3 | **30.8** |
| *Histone deacetylase 1 (HDAC1)* | 3.9 | 3.5 | **36.3** | 0.6 |
| *Histone deacetylase 3 (HDAC3)* | 0.3 | **11.3** | 5.8 | 3.3 |
| *Histone deacetylase 6 (HDAC6)* | 3.7 | 0.1 | 2.0 | 2.8 |

**Table S6:** **DNA-methylation gene contribution (5) of four-month-old juveniles based on the scores of two extracted principle coordinates.** Variance summaries are listed for the two principle components of the Between Class Analysis (BCA) (Inertia %) and contributions of each gene (gene contribution %) on the respective principle components (Axis1 and Axis2). Genes with a contribution of above 25 % summed average contribution, were considered as important genes which added the highest variance to the dimensional space and are marked in bold letters.

|  | **F0-bacteria** | | **F1-bacteria** | |
| --- | --- | --- | --- | --- |
|  | **Axis 1** | **Axis 2** | **Axis 1** | **Axis 2** |
| Inertia (Variance) % | 86.6 | 13.44 | 91.9 | 8.1 |
| *Gene Contribution %* | Contribution % | | Contribution % | |
| *DNA-methyltransferase 1* | 5.4 | 9.86 | 1.2 | 0.7 |
| *DNA-methyltransferase 3a* | **61** | 0.01 | **26.7** | **34.1** |
| *DNA-methyltransferase 3b* | **18.4** | **50.8** | **49.1** | 0.7 |
| *N6admet- methyltransferase* | 11.7 | **36** | **12.7** | **52.1** |
| *HemK2-methyltransferase* | 3.46 | 3.41 | 10.4 | 12.3 |

**Table S7. Linear Mixed effect model to test for F0-bacteria effects in time of maturation of adult pipefish males and clutch size of six-month-old F1-offspring.**

| **Maturity time** |  | **numDF** | **denDF** | **F-value** | **p-value** |
| --- | --- | --- | --- | --- | --- |
|  | (Intercept) | 1 | 126 | 29648.78 | <.0001 |
|  | F0-bacteria | 1 | 7 | **325.0** | **<.0001** |
|  |  | | | | |
|  |  | **numDF** | **denDF** | **F-value** | **p-value** |
| **Clutch size** | (Intercept) | 1 | 15 | 204.80 | <.0001 |
|  | F0-bacteria | 1 | 7 | **7.95** | **0.0257** |

**Table S8. Correlation analysis between immune genes and monocyte and lymphocyte count measurements from four-month-old juveniles.** By using a Pearson correlation matrix, each single gene (−∆Ct-values) was correlated with each immune cell measurement (Monocyte, Lymphocyte counts) in the head kidney (hk) and blood.

| **Monocytes head kidney** | **Monocytes blood** | **Lymphocytes hk & blood** |
| --- | --- | --- |
| **Lectin protein II** | **Lectin protein I** | **Complement component 1** |
| R^2^=0.26**, 0.0138 | R^2^=0.28**,0.038 | R^2^=-0.25*,0.016; -0.28**,0.007 |
| **Complement component 3** | **Complement component 3** | **HIVEP3** |
| R2=0.35***, <0.001 | R^2^=0.23***,0.0109 | R^2^=-0.23*,0.031 |
| **Interferon** | **Complement component 1** |  |
| R^2^=0.25**, 0.019 | R^2^=0.34***,<0.001 |  |
| **Peptidoglycan** | **Ik-cytokine** |  |
| R^2^=0.30**,0.004 | R^2^=0.23*,0.029 |  |
| **Tyroproteinkinase** | **Lymphantigen 75** |  |
| R^2^=0.23*,0.032 | R^2^=-0,22*, 0.038 |  |
|  | | |
